# The Future of Protozoan Infection Research: 3D Cell Culture and beyond

**DOI:** 10.1021/acsinfecdis.6c00244

**Published:** 2026-05-26

**Authors:** Sarah Beatriz de Fucio, Abel Sana, Beatriz Marques, Fernanda Araujo, Nusrat Sattar, Julia Charleaux, Jameel Inal, Marcel Ramirez, Izadora Volpato Rossi

**Affiliations:** † Postgraduate Program in Cellular and Molecular Biology, Federal University of Paraná, Curitiba 81531-970, Brazil; ‡ Postgraduate Program in Microbiology, Parasitology and Pathology, Federal University of Paraná, Curitiba 81531-980, Brazil; § Postgraduate Program in Biosciences and Biotechnology, Carlos Chagas Institute, Fundação Oswaldo Cruz, (FIOCRUZ-PR), Curitiba 81350-010, Brazil; ∥ School of Human Sciences, Cell Communication in Disease Pathology, 4904 London Metropolitan University, London N7 8DB, U.K.; ⊥ University of Hertfordshire School of Health, Medicine and Life Sciences, University of Hertfordshire, Hatfield AL10 9AB, U.K.; # Carlos Chagas Institute, Fundação Oswaldo Cruz, (FIOCRUZ-PR), Curitiba 81350-010, Brazil; ¶ Department of Immunology, Parasitology and Pathology, State University of Londrina, Londrina 86057-970, Brazil

**Keywords:** protozoan infections, three-dimensional cell culture, organoids, spheroids, microfluidics, organ-on-a-chip, host–parasite interactions, advanced in vitro models

## Abstract

Protozoan infections remain a major challenge to human health, owing to the substantial impact these parasites exert on their hosts. In vitro culture systems are fundamental for elucidating the host–parasite interactions underlying these infections; however, traditional two-dimensional models are often limited and fail to recapitulate key aspects of human tissue architecture and function. In this context, advanced in vitro cell culture models have emerged as powerful tools, offering increased morphofunctional resemblance to human tissues. This review critically examines recent studies employing advanced platforms, including Transwell systems, spheroids, organoids, scaffolds and biomaterials, microfluidic devices, and organ-on-chip technologies. Collectively, these approaches enable the modeling of diverse human tissues, such as the intestine, brain, heart, and vascular compartments, and support the investigation of multiple pathogenic protozoa, including *Trypanosoma cruzi*, *Plasmodium* spp., and *Giardia duodenalis*. By integrating advanced in vitro models into protozoan infection research, it is possible to achieve a more faithful representation of the human host environment, simulating infection processes and to foster the development of more effective and targeted pharmacological interventions.

## Graphical abstract

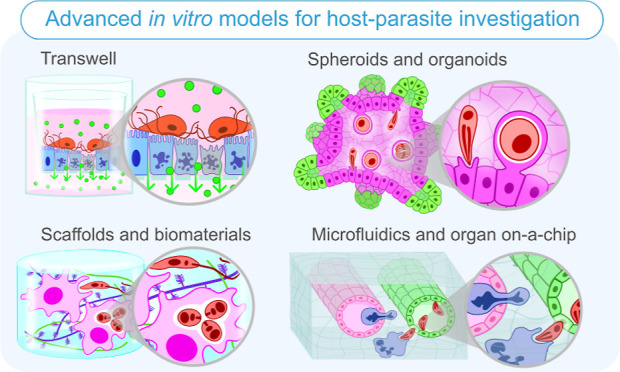

## 1 Introduction

Pathogenic protozoa comprise a polyphyletic group of unicellular eukaryotes that pose a major threat to global public health, accounting for high levels of morbidity and mortality.^1^ These infections are predominantly endemic in tropical and subtropical regions, affecting more than one billion people; however, their incidence has increased in developed countries as a result of climate change, global migration, and intensified international travel.^2^ Malaria alone causes approximately 170–174 million cases and more than 700,000 deaths annually worldwide, remaining one of the leading causes of infectious disease–related mortality in endemic regions. Similarly, leishmaniases account for an estimated 0.9–1.4 million new cases annually, while Chagas disease affects millions of individuals in Latin America, with high chronic prevalence and severe cardiovascular impact. In addition, enteric protozoan infections caused by organisms such as*Giardia duodenalis* and *Cryptosporidium*spp. contribute to approximately 7.5% of global diarrheal cases, according to epidemiological meta-analyses.^2,3^

Despite their substantial burden, many of these diseases are classified as neglected tropical diseases, reflecting limited investment in the development of new strategies for prevention, diagnosis, and treatment.^4^ From a biological standpoint, pathogenic protozoa exhibit complex life cycles, high genetic diversity, and efficient mechanisms of infection and immune evasion, frequently involving multiple developmental stages (Table 1; Figure 1). Together, these characteristics limit our understanding of protozoan pathogenesis and host–parasite interactions and significantly hamper the development of effective disease control measures.

**Table 1:** Summary of the Main Diseases Caused by Parasitic Protozoa and Relevant Aspects of Their Biology and Pathogenesis^a^

disease	protozoa species	main systems/organs involved	transmission	requirement of one (monoxenous) or more hosts (heteroxenous)	intra/extracellular	stages in human host
Chagas disease	*Trypanosoma cruzi*	Cardiac; GIT; Muscular; Blood	Vetorial (Triatominae–kissing bug)	Heteroxenic	Intracellular	Trypomastigotes; Amastigotes
Leishmaniasis	*Leishmania* spp.	Skin; mucosa; liver; spleen; bone marrow	Vectorial (sandfly–Phlebotominae)	Heteroxenic	Intracellular	Promastigotes; Amastigotes
African trypanosomiasis (Sleeping sickness)	*Trypanosoma brucei*	Blood; Lymphatic; CNS	Vectorial (tsetse fly–*Glossina*)	Heteroxenic	Extracellular	Trypomastigotes
Malaria	*Plasmodium* spp.	Liver; Blood; CNS	Vectorial (*Anopheles*)	Heteroxenic	Intracellular	Sporozoite; Merozoite; Gametocyte; Trophozoite; Schizont
Toxoplasmosis	*Toxoplasma gondii*	CNS; Ocular; Muscular; Placenta	Oral; Vertical	Heteroxenic	Intracellular	Tachyzoite; Bradyzoite
Giardiasis	*Giardia duodenalis*	GIT	Oral	Monoxenic	Extracellular	Trophozoite; Cyst
Cryptosporidiosis	*Cryptosporidium* spp.	GIT	Oral	Monoxenic	Intracellular	Oocyst; Sporozoite, Trophozoite
Amoebiasis	*Entamoeba histolytica*	GIT	Oral	Monoxenic	Extracellular	Cyst, trophozoite
Acanthamoebiasis	*Acanthamoeba* spp.	Ocular; CNS	Dermic; Inhalation	Monoxenic	Extracellular	Cyst, trophozoite

a GIT (Gastrointestinal tract); CNS (Central nervous system).

**Figure 1:**
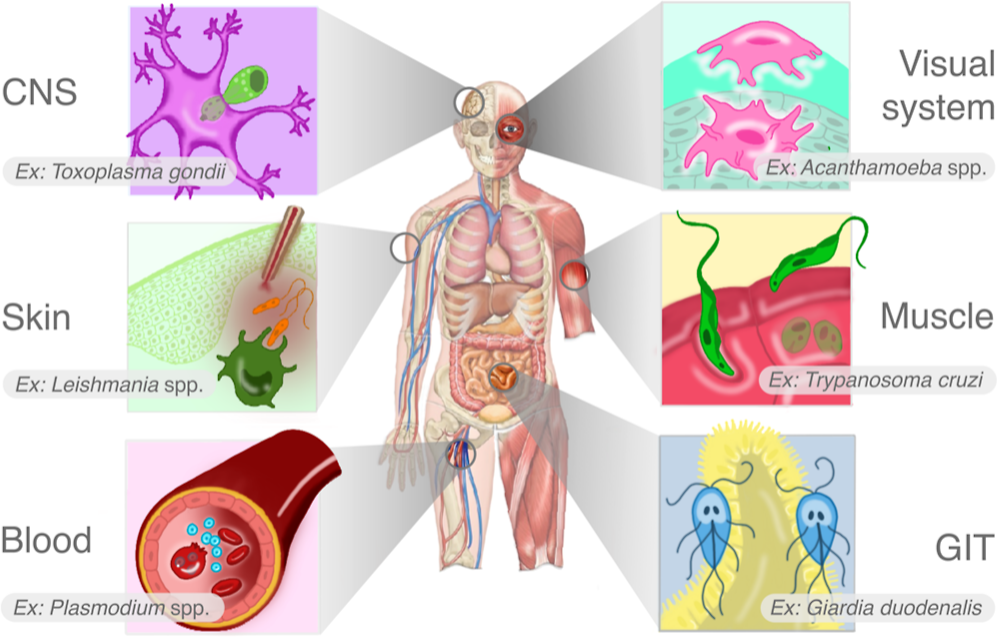
Schematic representation of the tissue tropism of pathogenic protozoa in humans. Representative examples include *Toxoplasma gondii* in the central nervous system (CNS), *Leishmania* spp. in the skin, *Plasmodium* spp. in the bloodstream, *Acanthamoeba* spp. in the visual system, *Trypanosoma cruzi* in muscle tissue, and *Giardia duodenalis* in the gastrointestinal tract (GIT).

In this context, research aimed at elucidating protozoan life cycles, pathogenic mechanisms, and drug susceptibility is essential not only for clarifying the tissue and functional damage inflicted on the host, but also for supporting the development of more effective and safer therapeutic strategies. Conventional two-dimensional (2D) in vitro models, such as cell monolayers, represented a major advance in early studies of host–parasite interactions, enabling analyses of adhesion, cellular invasion, and drug uptake and efficacy.^5^ However, these systems do not adequately reproduce the three-dimensional complexity of tissues, nutrient gradients, mucosal barriers, or spatially organized immune responses observed in complex organisms, which may lead to underestimation of relevant pathophysiological mechanisms and limited translational reproducibility in clinical trials.^6^ These limitations reinforce the need for more biomimetic experimental platforms.

Importantly, this problem is not merely an academic consideration but is also reflected in the priorities of international funding agencies such as the world health organization (WHO), creating the WHO roadmap for Neglected Tropical Diseases (2021–2030),^7^ which establish a strategic global framework to eliminate, eradicate or control 21 neglected tropical diseases, and prioritizes research and innovation; and the National Institutes of Health (NIH) announced an initiative in April 2025 to prioritize research technologies based on human-relevant models. This initiative aims to reduce animal use while promoting innovative approaches such as organoids, organ-on-chip systems, other advanced in vitro platforms, computational models, and real-world data that offer greater biological relevance to human health and closer alignment with clinical reality compared with traditional in vivo models.

Given the global impact of infections caused by pathogenic protozoa and the limitations of conventional experimental systems, this review aims to systematize the main advances in in vitro models used to study protozoan infections, with emphasis on Transwell cultures, spheroids, organoids, scaffolds, biomaterials, microfluidic technologies, and organ-on-chip systems (Supporting Information Table S1). We also have a purpose to stimulate the use and definition of new culture systems and methods to better represent parasite host cell interactions. In addition, we critically discuss the potential, limitations, and challenges of these models, highlighting their translational relevance for understanding host–parasite interactions and for the development of novel therapeutic approaches.

## 2 Traditional in Vitro Models for Studying Protozoa

A substantial proportion of the current knowledge on parasitic infections derives from studies employing traditional cell culture models. Although for many years these approaches have been widely used because of their simplicity, lower cost, and high reproducibility, growing evidence indicates that two-dimensional (2D) systems fail to faithfully recapitulate complex physiological environments.^6^ In 2D cultures, cells are constrained to an artificial monolayer organization that poorly reflects tissue architecture and function. In contrast, recent advances in in vitro cell culture models, encompassing not only three-dimensional (3D) systems but also compartmentalized, dynamic, and multicellular platforms, have begun to reshape current perspectives on the most appropriate systems for reproducing infections in vitro. These models better reproduce key features of native tissues, including spatial organization, mechanical cues, and microenvironmental complexity. Consequently, 2D systems exhibit inherent limitations, such as restricted cellular differentiation and function, as well as oversimplified microenvironments that fail to capture complex cell–cell and cell–extracellular matrix (ECM) interactions.^6,8^

Accordingly, recent evidence demonstrates that host–parasite interactions are significantly influenced by the cellular model employed. In the case of*Toxoplasma gondii*, for example, infected cells cultured within three-dimensional collagen matrices exhibit marked alterations in parasite replication, egress, and intravacuolar organization when compared with conventional bidimensional models.^9^ Similarly, three-dimensional models have revealed differences in*Trypanosoma cruzi* infection dynamics, as trophoblastic cells cultured as spheroids display reduced susceptibility to the parasite relative to 2D cultures.^10^ Importantly, limitations of 2D systems extend beyond the study of host–parasite interactions and also affect our understanding of antiparasitic drug activity. For instance, O’Keeffe et al. (2020) evaluated antileishmanial drug efficacy using complex in vitro platforms, including perfused systems and 3D cell cultures, in comparison with traditional models.^11^ Their findings demonstrated that assay complexity significantly impacts antileishmanial drug activity, suggesting that, although these results may not yet justify a complete shift in routine screening assays, advanced in vitro models offer valuable opportunities for pharmacokinetic and pharmacodynamic (PK/PD) studies under more physiologically relevant conditions. There is a clear shift toward 3D systems, as they better represent tissue physiology and parasite-host cell interactions.

## 3 Next-Generation In Vitro Models for Protozoan Research

### 3.1 Barrier Models Using Transwell Systems

Transwell systems consist of permeable supports with porous membranes that divide a conventional cell culture well into two distinct compartments (Figure 2A). This apical–basal compartmentalization enables improved cellular interactions and supports experimental approaches that are not feasible in traditional bidimensional culture systems. Accordingly, Transwell methodologies have been widely applied to the analysis of drug absorption and permeability, immune cell migration across epithelial barriers, and parasite invasion of diverse tissues.^12–14^

**Figure 2:**
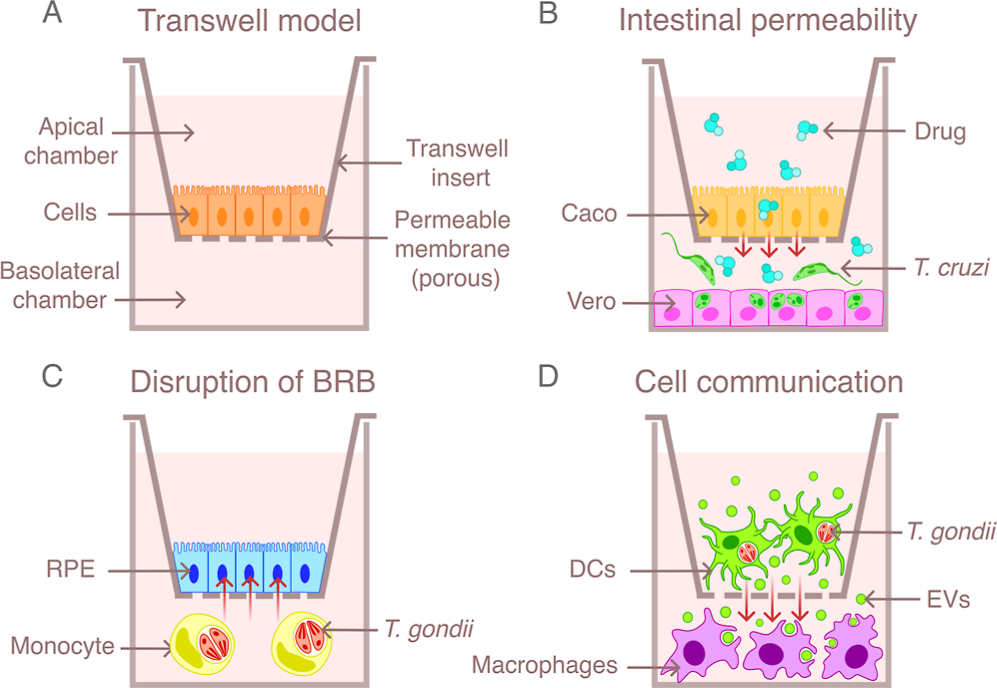
Transwell cell culture model and applications in the study of protozoan–host interaction. (A) Basic Transwell model containing an apical and basolateral chamber separated by a permeable porous membrane. (B) Model for studying intestinal permeability to drugs, using intestinal epithelial cells in the apical chamber and*T. cruzi*-infected cells in the lower layer. (C) Model of disruption of the blood-retinal barrier (BRB), using retinal pigment epithelial cells (RPE) in the upper chamber and monocytes infected with *T. gondii* in the lower layer. (D) Model for studying cell communication through extracellular vesicles (EVs), using dendritic cells (DCs) infected with*T. gondii* and macrophages as EV receptors.

**Figure 3:**
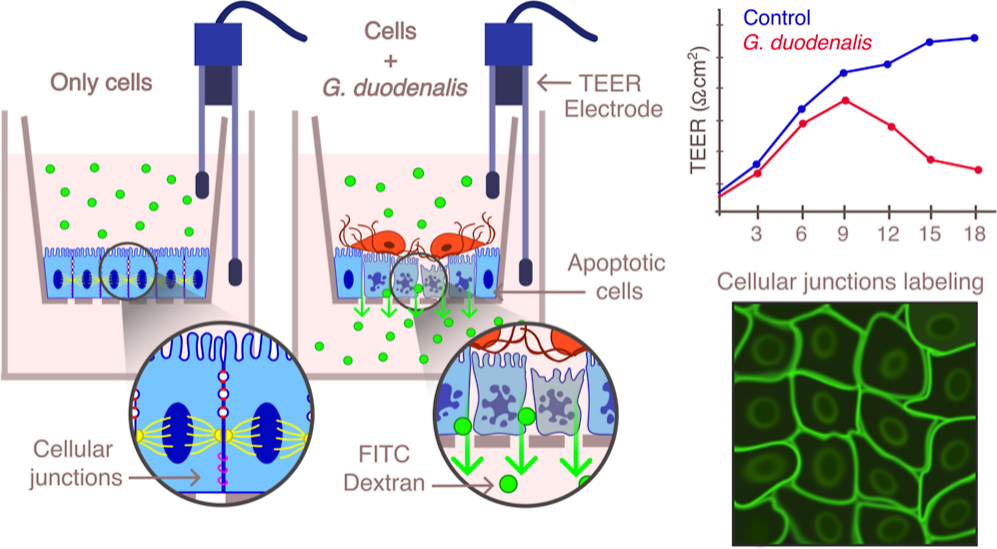
Schematic representation of the methods for checking cell barrier formation in Transwell, exemplified by an intestinal epithelium and the damage caused by*G. duodenalis*, leading to loss of intercellular junctions, epithelial disruption, and increased cell permeability.

Due to their technical simplicity and versatility, Transwell platforms can accommodate a wide range of cell lines, enabling the in vitro representation of multiple tissues and organs, including intestinal epithelium, lung, heart, and blood-associated compartments. The incorporation of more than one cell lineage within the same system further enhances model complexity, as cocultures (either within the same compartment or across opposing compartments) more closely recapitulate the morphology and physiology of the tissue of interest and have been widely used for over a decade.^15^

The intestinal epithelium is a primary site of infection for several protozoan parasites, making its faithful in vitro modeling essential for investigating parasite pathogenesis, host tissue damage, and therapeutic responses. As many of these processes cannot be adequately assessed in conventional bidimensional cultures, Transwell systems represent a particularly relevant platform for intestinal studies^16^ (detailed in Box [Boxed-text dbox1]). This approach has been extensively applied to the study of*G. duodenalis*, a protozoan that strongly adheres to the intestinal mucosa. Transwell-based intestinal models have been established using Caco-2 cells, coculture systems, or cells derived from human duodenal organoids (ODMs).^17–22^

1 **Methods for Validating Barrier Formation in Transwell**

Barrier formation in Transwell systems is based on the ability of epithelial or endothelial cells to adhere to the porous substrate, proliferate, and organize into a continuous, polarized monolayer capable of physically separating the apical and basolateral compartments. This process depends strongly on the establishment and maturation of cell junctions, especially tight junctions, adherens junctions, and desmosomes, which coordinate cell cohesion and control paracellular flux^31^ (Figure 3).

Assessment of barrier formation and integrity in Transwell systems can be performed using functional, morphological, and molecular approaches, which provide complementary information. Measurement of transepithelial/transendothelial electrical resistance (TEER) is a widely used functional technique based on quantifying resistance to ion flow across the cell monolayer; high TEER values indicate greater intercellular cohesion and better sealing of tight junctions, allowing continuous monitoring of barrier maturation over time, detecting the establishment and stability of tight junctions and monolayer integrity.^32–34^

However, baseline TEER values vary substantially across tissue types and cell models, meaning that absolute resistance values are not directly comparable between different epithelial or endothelial systems. Therefore, barrier disruption or reinforcement should be interpreted relative to tissue-specific reference ranges and complemented with additional permeability and structural readouts.

Paracellular permeability assays evaluate the transport of tracer molecules, such as fluorescent dextrans or radiolabeled compounds, from the apical to the basolateral compartment; the passage rate of these tracers, especially when using different molecular weights, provides a direct estimate of the selectivity and functional integrity of the barrier.^35,36^

Morphological analyses, in turn, include immunofluorescence and confocal microscopy, which allow visualization of monolayer organization and the subcellular localization of junctional proteins such as claudins, occludin, and ZO-1, demonstrating junction continuity and polarization. Complementary molecular and biochemical methods, such as qPCR and Western blot, are employed to quantify gene and protein expression of junctional components, enabling correlation of functional barrier changes with alterations in levels and regulation patterns of these proteins during the cell differentiation process.^37,38^

Beyond *G. duodenalis*, intestinal Transwell models have also been employed to investigate infections by *Cryptosporidium parvum*, a parasite classically associated with the gastrointestinal tract, and *T. gondii*, whose life cycle transiently involves the intestinal epithelium.^20,23^ Evaluation of intestinal epithelia formed in a 3D culture in Transwell systems commonly includes measurements of transepithelial electrical resistance (TEER), permeability assays using fluorescent tracers such as FITC-dextran, immunofluorescence analysis of intercellular junctions, and molecular analyses by RT-qPCR and/or Western blotting^18–22^ (detailed in Box [Boxed-text dbox1]-fo). For instance, Holthaus et al. (2021) demonstrated that *G. duodenalis*, but not *T. gondii*, disrupts membrane permeability, epithelial integrity, and the transcriptional abundance of tight junction components in intestinal cells cultured in Transwell systems.^20^ Similar findings were reported for *Cryptosporidium* infection, in which parasite-induced disruption of tight and adherens junctions leads to barrier dysfunction and contributes to the diarrheal manifestations of cryptosporidiosis.^23^

For the investigation of parasites that migrate through the myocardium, such as *T. cruzi*, the use of representative cardiac cell models is essential. Hernández et al. (2016) proposed a Transwell-based system using HL-1 cells and primary mouse cardiomyocytes to evaluate the cardioprotective effects of curcumin, which reduced *T. cruzi*-induced inflammation and attenuated infection-associated cardiac damage.^24^ In a different Chagas disease-related context, a Transwell model was employed to assess intestinal absorption and downstream efficacy of a compound against *T. cruzi*.^12^ In this system, treatment was applied to the apical compartment containing Caco-2 intestinal cells and subsequently reached the lower compartment harboring *T. cruzi*-infected Vero cells. This approach demonstrated effective intestinal permeation of the compound and inhibition of infection in the lower compartment (Figure 2B). Additionally, Transwell platforms have been used to simulate *T. cruzi* migration across the gastric mucus layer, mimicking oral infection routes. Assays using mucin-coated filters revealed strain-dependent differences in parasite migratory capacity.^25^

The brain is an exceptionally complex organ that can be modeled using different Transwell-based configurations. To investigate direct interactions of *T. gondii* within the central nervous system, Tao et al. (2023) employed an indirect coculture system using murine hippocampal neuronal cells (HT22) and microglial cells (BV2) during infection.^26^ In contrast, the model developed by Wang et al. (2025) was designed to examine the role of microglia in the recruitment and activation of CD8^+^ T cells during cerebral malaria.^2^ In this system, primary cortical microglia were exposed to *Plasmodium berghei*-infected erythrocytes and CD8^+^ T cells, resulting in the recruitment and activation of these immune cells.

The blood-retinal barrier (BRB) is a key target of *T. gondii* during ocular toxoplasmosis. Song et al. (2017) developed a Transwell-based model to simulate immune cell migration across this barrier.^27^ The system consisted of *T. gondii*-infected monocytic cells (THP-1) and human retinal pigment epithelial cells (ARPE-19) seeded on the lower surface of the Transwell membrane, while additional monocytic cells were added to the lower compartment to assess migration toward the apical side (Figure 2C). In this model, parameters such as TEER, cytokine profiles, and tight junction integrity were analyzed by immunofluorescence during parasite invasion. A similar approach was later used to investigate the migration of *T. gondii*-infected neutrophils across a barrier formed by human retinal pigment epithelial cells, either primary cells isolated from human donors or the ARPE-19 cell line.^28^

Immune cells are profoundly affected during protozoan infections, either through direct invasion or through their recruitment to infection sites. In this context, Jiang et al. (2022) demonstrated, using a Transwell-based assay, that *T. gondii*-infected dendritic cells secrete exosomes enriched in miR-155-5p that modulate anti-infective responses in macrophages,^29^ highlighting the role of extracellular vesicle-mediated paracrine communication (Figure 2D). Similarly, Reddy et al. (2021) developed a Transwell-based interaction system between *Plasmodium falciparum* and human B cells to assess the impact of direct cell–cell contact on parasite growth and immune cell proliferation.^30^ Boström et al. (2017) further employed functional assays to analyze neutrophil alterations and *P. falciparum*-mediated chemotaxis in the context of pregnancy-associated malaria.^13^ Collectively, these studies support the utility of Transwell-based models for the controlled analysis of immune–parasite interactions.

### 3.2 Spheroids and Organoids

Building on the limitations of Transwell and conventional 3D cultures, more advanced models such as spheroids and organoids have emerged to better reproduce tissue organization and physiological complexity. Spheroids are spherical cellular aggregates with a self-organized nature (Figure 4A), formed in an environment that prevents adhesion to a surface^39,40^ (detailed in Box [Boxed-text dbox2]). These structures are formed when cell–cell interactions predominate over cell-ECM interactions. The adhesive properties of cells play an essential role in their formation, which begins with cell aggregation mediated by interactions between integrins present on the cell membrane and ECM components released by the cells themselves during culture or artificially introduced.^39,41^ ECM fibers containing RGD (arginine–glycine-aspartate) motifs are recognized by integrins on the cell membrane. This interaction allows dispersed cells to aggregate more rapidly. Subsequently, there is an increase in cadherin synthesis, which accumulate on the cell surface and interact with each other (homophilic cadherin–cadherin interactions), leading to the gradual formation of a compact spheroid structure.^39,41^ Therefore, spheroid formation involves: (I) aggregation of dispersed cells, (II) accumulation of cadherins on the cell surface, and (III) homophilic interactions of cadherins between neighboring cells.^41^

**Figure 4:**
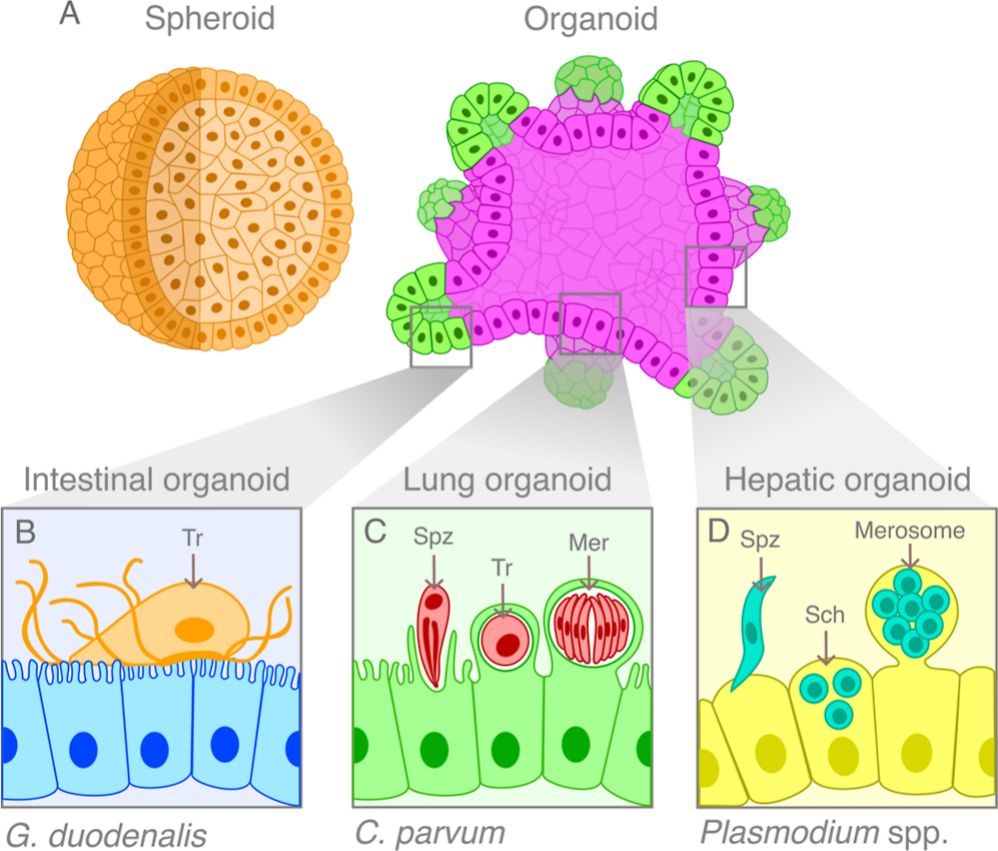
Cellular models of spheroids and organoids: applications in parasite-host interaction studies (A) Schematic representation of spheroid (left) and organoid (right). (B) Intestinal organoid model for studying the adhesion of *G. duodenalis* trophozoites (Tr) to the intestinal epithelium. (C) Lung organoid used to study the cell cycle of *C. parvum* (Spz sporozoite; Tr trophozoite; Mer merozoite). (D) Liver organoid for studying *Plasmodium* spp. (Spz sporozoite; Sch schizont).

2 **Come Together: Methods for Spheroid and Organoid Formation and Limitations**

The generation of spheroids is primarily based on strategies that promote controlled cell aggregation and the formation of stable three-dimensional structures. Initially, cells are dissociated into a single-cell suspension and seeded into systems that minimize surface adhesion, such as low-adhesion plates, U-bottom microplates, or the hanging drop method, in which droplets containing a defined number of cells are inverted to allow aggregation by gravity.^61^ Initial cell density is a critical factor, as it directly influences spheroid size and compaction. During culture, cell–cell interactions mediated by cadherins and cytoskeletal reorganization promote progressive structural compaction, while the limited diffusion of oxygen and nutrients establishes physiologically relevant gradients within the aggregate.

Organoid formation involves more complex and highly controlled steps, beginning with the expansion of pluripotent stem cells or adult stem cells isolated from tissue-specific sources. After dissociation, these cells are embedded in a three-dimensional ECM, typically in the form of hydrogel droplets, which provide both physical support and essential biochemical signals for self-organization. Defined culture media supplemented sequentially with growth factors, signaling pathway inhibitors, and morphogens are then applied according to tissue-specific protocols. Temporal modulation of these conditions directs cell differentiation, epithelial polarization, and the formation of functional domains, resulting in structures that exhibit architecture similar to that of the tissue of origin.^62^

Organoid models are one of the most expensive and resource-intensive in vitro systems, requiring expensive growth factors-rich media, ECM gels, specialized infrastructure, and long culture times. Additional costs arise from long-term maintenance, batch variability, and quality control. Beyond financial demands, organoid research faces ethical, regulatory, and logistical constraints, as it often relies on patient-derived tissues, requiring ethics approval and informed consent, which can delay experiments. Studies also depend on patient availability and sample quality, introducing variability and limiting scalability compared with immortalized cell lines commonly used in 2D and some 3D models. Rigorous control of the microenvironment, including matrix composition, soluble factor concentration, and culture duration, is essential to ensure reproducibility and proper organoid maturation.

Since the original introduction of spheroids in the early 1970s by Sutherland and Durand, several spheroid models have been developed and have become one of the most popular methods for three-dimensional cell culture.^42,43^ In the context of parasitic diseases, spheroids have been used to study host interactions with *Plasmodium vivax*,^44^ *T. cruzi*,^45–49^ and *T. gondii*.^50^ This approach has enabled significant advances in understanding the complexity of parasitic diseases in in vitro studies.

Although cellular spheroids are more complex and more accurately mimic cell–cell interactions than 2D cell cultures, they are not able to fully represent the complexity of the tissue microenvironment. In this context, organoid technology has emerged as an in vitro model system to overcome some of the challenges associated with 2D and spheroid models. An organoid is, in simple terms, a three-dimensional multicellular tissue produced in vitro that resembles an in vivo organ in both structure and function (Figure 4A). Organoids have the ability to self-differentiate and to exhibit properties and functions similar to those of human organs.^43,51^ Organoids are derived from pluripotent or adult stem cells (PSC and ASC, respectively), differentiated by sequential growth factor signaling that recapitulates key aspects of embryonic development.^52^

In the context of diseases caused by protozoa, several organoid models have already been established. Seo and colleagues (2020)^53^ developed human brain organoids as an in vitro model of infection by *T. gondii*. The authors demonstrated that the tachyzoite forms of *T. gondii* were able to infect the organoids after 4 h of incubation and to differentiate into the bradyzoite stage within the organoids. In addition, they observed that the parasite preferentially infected neurons, astrocytes, and oligodendrocytes, but not radial glial cells, and that *T. gondii* remained virulent in infected organoids. Brain organoids were also used by Chandrasegaran and colleagues (2023),^54^ who developed a coculture system using human cortical brain organoids derived from induced pluripotent stem cells (iPSCs) to study the interaction between *Trypanosoma brucei* and the host. By analyzing the response of the organoids to *T. brucei* infection, the authors observed transcriptional changes, including the upregulation of genes associated with blood vessel differentiation, innate immune responses, and chemotaxis.

Intestinal organoids have been used as in vitro models of infection by *T. gondii* ^55^ and *C. parvum*.^56–58^ In the case of *T. gondii*, intestinal organoids are particularly relevant models, as the intestine is the site of the parasite’s sexual reproduction (gamete formation). Cancela and colleagues^59^ investigated *T. gondii* infections in murine intestinal organoids, in which immunofluorescence analysis of the tachyzoite surface antigen SAG1 confirmed the intracellular localization of the parasite and its active replication within the organoids. However, higher infection efficiency was observed in 2D monolayer cultures compared with organoid cultures. This finding suggests that multiple factors present in organoid cultures, but absent in 2D monolayers, may influence the success of infection. In the case of *C. parvum*, Heo and colleagues^57^ introduced parasite oocysts into the lumen of human small intestinal organoids by microinjection. Using qPCR targeting *C. parvum* 18S rRNA, the authors observed that, in both expanding and differentiated organoids, rRNA levels increased by several orders of magnitude 24 h after infection, demonstrating that the parasites are able to propagate within the organoids. In addition, the authors reported that *C. parvum* can complete its entire life cycle within intestinal organoids.

Intestinal organoids, as models of infection by protozoa that colonize the intestine, have also been employed to study the interaction between *G. duodenalis*, the etiological agent of giardiasis, and the host. Infection by *G. duodenalis* can cause alterations in the function and integrity of the intestinal barrier. These changes are not fully captured by conventional cell line-based models, such as Caco-2 cells derived from colon carcinoma, making intestinal organoids particularly relevant models for investigating parasite–epithelium interactions. Epithelia derived from human organoids preserve cellular polarity, intercellular junctions, and barrier functions, enabling detailed investigation of the damage induced by *G. duodenalis* (Figure 4B). Holthaus et al.^21^ explored the mechanisms of epithelial barrier dysfunction induced by *G. duodenalis* in organoid-derived epithelia and observed alterations in barrier integrity, as indicated by a dose- and time-dependent decrease in transepithelial electrical resistance (TEER).

Heo et al.^57^ also used lung organoids composed of basal cells, ciliated cells, goblet cells, and Club cells to model *C. parvum* infection (Figure 4C). Quantification of 18S rRNA showed that the parasite increased dramatically 24 h after injection, similar to the growth observed in small intestinal organoids. Indirect immunofluorescence (IIF) using antibodies specific for zoites revealed the development of both asexual (meront I) and sexual (microgamont) stages. Furthermore, transmission electron microscopy (TEM) demonstrated that the parasite was able to infect both secretory and nonsecretory cells in lung organoids.

Hepatic organoids have been used to study the interaction of *P. falciparum* with hepatocytes (Figure 4D). Parasite development in the liver represents the initial stage of the life cycle in the human host. Human hepatic organoids offer advantages over two-dimensional hepatocyte cultures, as they preserve cellular heterogeneity, three-dimensional organization, and liver-specific metabolic functions. In this context, Yang and colleagues^51^ (worked with four human fetal hepatocyte organoid lines (KU1, KK2, KK3, K1FM) and observed that these organoids are susceptible to *P. falciparum* infection and maintain the mature liver schizont stage, as evidenced by MSP-1 expression in late hepatic stages. Moreover, the parasite transcriptome of human tissue infected organoids showed upregulation of several markers specific to the hepatic stage of *P. falciparum*, including CSP, LISP1, and SLARP. Mellin and Boddey (2020)^60^ also demonstrated that hepatic organoids support sporozoite invasion and parasite development within cells.

Despite their sophisticated and clear advantages, organoid models are expensive, rely on limited access to primary tissues, show variability that complicates standardization and raise ethical issues related to human tissue sourcing and consent. Both spheroid and organoid technologies represent significant advances in the in vitro study of parasitic diseases. However, these models lack general physiological processes that influence the function of tissues in vivo. The absence of a circulatory system is a limiting factor, as cells located at the center of a spheroid or organoid do not receive nutrients adequately and do not properly eliminate metabolic waste.^43^ In order to overcome these limitations and more closely mimic the in vivo environment, microfluidic systems are being developed. Despite these limitations, the use of organoids to study protozoan–host interactions has allowed researchers to approach as closely as possible the in vivo reality of these interactions/infections.

### 3.3 Scaffolds and Biomaterials

A scaffold is a three-dimensional porous structure composed of a biocompatible substance that functions as a template for tissue regeneration while cells are inserted in a specified biomechanical environment (Figure 5A). It functions as a transient ECM that mimics the mechanical and biological features of natural tissue to facilitate incorporation and the development of new tissue, especially in soft tissues including cartilage, skeletal muscles, skin, and ligaments, and hard tissues comprising bone and teeth.^63–65^ They are essential components in growth, proliferation, development of new tissues, and drug delivery.^66^

**Figure 5:**
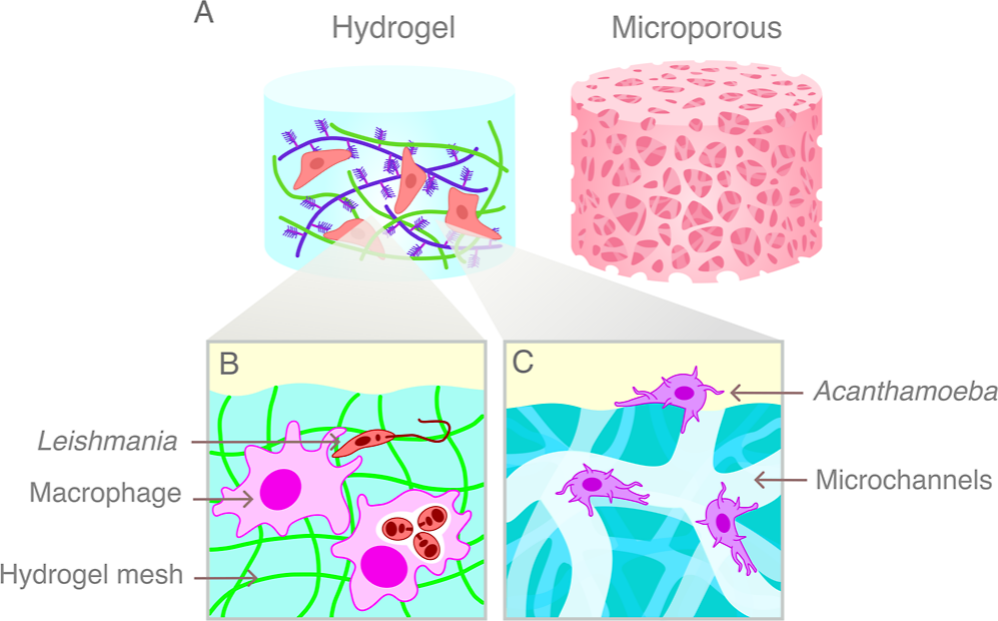
Schematic representation of biomaterials and applications in parasite-host interaction. (A) Schematic representation of hydrogels and microporous scaffolds. (B) Application of hydrogels that act against *Leishmania* but are not toxic to macrophages. (C) Hydrogels produced containing microtunnels that can capture *Acanthamoeba*.

Biomaterials are important for the preparation of scaffolds. Biomaterial for scaffolds includes natural and synthetic biomaterials. Natural biomaterials further categorized into two classes include protein origin (silk, collagen, fibrin, gelatin, etc.) and polysaccharide origin (cellulose, chitin, starch, dextran, hyaluronan, alginate, agarose, chitosan, alginate, etc.).^67^ Whereas synthetic biomaterials include polymers (polyethylene glycol, polyglycolide, poly lactic-co-glycolic acid, poly-D,l-lactide, poly e-caprolactone, etc.)^68^ and ceramic biomaterials (alumina, zirconia, sintered HA, or β tricalcium phosphate, tetracalcium phosphate, hydroxyapatite, bioactive glass, calcium phosphate, etc.).^69,70^ The development of novel substances or the modification of the composition and microstructure of existing substances for tissue engineering remains a major concern of recent research. Some details regarding the fabrication of scaffolds and the use of biomaterials are described in Box [Boxed-text dbox3]. The scaffolds should include characteristics of cell proliferation, tissue differentiation, and mechanical optimization properties of regenerating tissues.^71^ The main advantage of biomaterial based scaffolds is that they allow researchers to control tissue shape, structure, and stiffness, whereas spheroids, organoids, and other 3D models rely on self-organization with less control and reproducibility.

3 **3D Bioprinting for Scaffold Engineering and Pathogen–Host Interactions**

3D bioprinting and related three-dimensional culture technologies provide powerful tools to study interactions between pathogens and eukaryotic host cells in physiologically relevant environments. By enabling precise control over tissue architecture, cell polarity, and microenvironmental conditions, these systems more accurately reproduce host barriers and infection dynamics than conventional 2D models.^85,86^

Extrusion-based 3D bioprinting and hydrogel-based platforms allow the fabrication of structured epithelial and multicellular constructs that mimic native tissue organization. These models support controlled investigation of pathogen adhesion, invasion, replication, and dissemination across host barriers, while remaining scalable and compatible with multiple cell types.^87^

Light-based bioprinting technologies, such as stereolithography (SLA) and digital light processing (DLP), enable high-resolution fabrication of complex host–pathogen interfaces. Tunable photocurable hydrogels permit modulation of matrix stiffness, permeability, and receptor presentation, although material biocompatibility and phototoxicity remain technical challenges.^88^

Microstructured and droplet-based approaches, together with multimaterial and hybrid 3D bioprinting, allow spatial patterning of epithelial cells, immune cells, ECM components, and live pathogens within a single construct, increasing biological realism. These systems are increasingly combined with organ-on-a-chip and microfluidic platforms to simulate flow, shear stress, and compartmentalized infection, critical for modeling intestinal, respiratory, and vascular pathogen interactions.

Electrospun nanofibers are widely used to create biomimetic 3D scaffolds that mimic the extracellular matrix (ECM), offering high porosity for cell infiltration, nutrient exchange, and customizable mechanical properties to support tissue regeneration. Fiber-based scaffolds can be engineered to support host tissue cells, so stimulating in vivo conditions for parasite infection models.^89,90^

Despite their promise, challenges remain in maintaining long-term tissue viability, incorporating immune complexity, standardizing infection readouts, and scaling for high-throughput applications. Continued innovation in biomaterials, imaging, and computational modeling is expected to expand the impact of 3D bioprinting in host–pathogen research.^91^

The principles of scaffolds depend on potential biological qualities, such as biocompatibility, which provide cell adhesion, vascularization, supply of oxygen and nutrients, and molecular signaling systems.^72^ Similarly, bioactivated scaffolds release cytokines, growth factors and ECM also promote angiogenesis, ensure adequate blood supply, and influence cell differentiation.^73,74^ Whereas, the biodegradable scaffolds have the capacity to stimulate cell invasion, adhesion, cell proliferation and also produce their own ECM.^75^ Furthermore, scaffolds exhibit tissue-specific mechanical qualities, including elastic modulus, tensile strength, viscoelasticity, stiffness, and porosity.^76–78^ Porosity plays an important role in scaffold design, impacting not only the material’s mechanical and biological characteristics but also the scaffold’s physio-thermal properties and internal transport dynamics.^69,79^

Biomaterials have also been seen as platforms capable of interacting directly with parasites, influencing their survival, mobility, and infectivity. Previous research indicated nucleoside-based biocompatible hydrogels exhibited no cytotoxic effect on the macrophage cell line, but a significant leishmanicidal response was produced against *Leishmania* major’s promastigotes and amastigotes, which could be used as a topical treatment for cutaneous leishmaniasis^80^ (Figure 5B). Hydrogel structures have also proven useful for capturing *Acanthamoeba castellanii*, an amoeba that is difficult to eliminate from the environment and tissues. The hydrogel structures contain a labyrinthine three-dimensional network of interconnected microchannels, in which the parasite is captured and can be removed from the incubation medium^81^ (Figure 5C). Similarly, due to great mechanical strength, exceptional stability, immense drug-loading capacity, and prolonged and sustained drug release, hydrogels and drug-loaded hydrogels are also utilized as antiparasitics, as already shown for helminths.^82–84^ The studies on the impact of scaffolds and tissue engineering against protozoan-related diseases are still very limited.

### 3.4 Microfluidics and Organ-on-a-Chip Systems

Microfluidics is a multidisciplinary branch of science that studies the behavior, control, and manipulation of fluids at the micrometer scale, employing microminiaturized devices with very small chambers and channels.^92^ This technology constitutes the foundational basis for the development of organ-on-a-chip (OOC) systems, which represent some of the most advanced biomimetic platforms currently available in experimental biomedicine.^93^ OOC devices employ interconnected networks of microchannels and microchambers (Figure 6A) that enable precise control of fluid flow, chemical gradients, mechanical forces, and cell–cell and cell-ECM interactions, thereby recreating key structural and functional features of human tissues and organs in vitro.^94^ The manufacturing characteristics and properties of the chips are described in Box [Boxed-text dbox4]. To date, multiple human organs have been modeled using microfluidic and OOC technologies, including skin, spleen, intestine, liver, brain, and eye,^95^ and applications in parasitology are emerging over the past five years.

**Figure 6:**
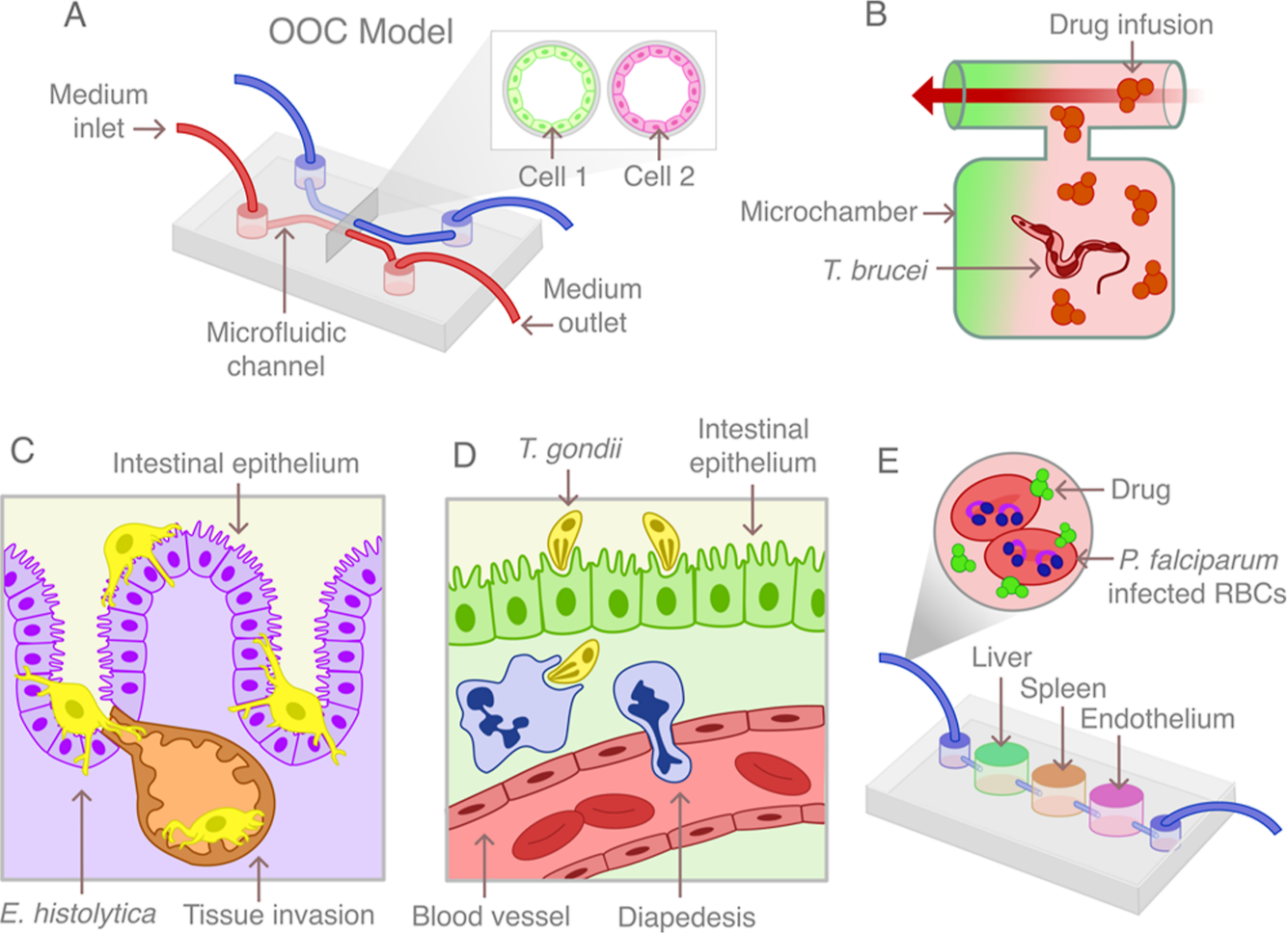
Representative diagram of organ-on-a-chip (OOC) and applications in parasite-host interaction. (A) Schematic representation of OOC. (B) Application of microfluidics in the study of anti-*T. brucei* drugs. (C) Intestinal OOC for the study of epithelial invasion and lesion formation by *E. histolytica*. (D) OOC model for reproducing the transendothelial migration of immune cells in *T. gondii* infection. (E) Multiorgan OOC for the study of the therapeutic action of antimalarial drugs.

4 **From Microfluidics to Microphysiology**

The development of an organ-on-a-chip begins with microfabrication of the chip structure, typically using techniques adapted from microelectronics, such as photolithography and soft lithography. The most commonly used material is PDMS (polydimethylsiloxane) due to its optical transparency, gas permeability, biocompatibility, and ease of molding.^111^ Microfluidic channels within the chip are usually designed at micrometer scale (tens to hundreds of micrometers), allowing the recreation of physiological microenvironments similar to capillary beds and tissue interstitial spaces. After molding, the PDMS layer is bonded to glass or another polymer surface to seal the device and create closed compartments suitable for cell culture under controlled conditions.

Fluid flow control is a critical feature of these systems. External pumps, such as syringe pumps or peristaltic pumps, are connected to the chip through microtubing to enable continuous perfusion of culture medium, mimicking blood or interstitial flow. Flow rates can be precisely adjusted to regulate shear stress, nutrient delivery, and metabolite removal.^112^ More advanced platforms integrate microvalves and automated controllers that allow generation of chemical gradients, pulsatile flow, and other dynamic conditions that more closely reproduce in vivo physiology.^113,114^ Additionally, many chips incorporate embedded sensors for real-time monitoring of parameters such as pH, dissolved oxygen, and transepithelial electrical resistance (TEER), which is particularly useful for assessing barrier formation and tissue functionality.^115^ This precise spatial, mechanical, and biochemical control makes organ-on-a-chip platforms powerful tools for disease modeling, toxicology testing, and drug development.

Early and relatively simple microfluidic systems have already contributed to fundamental insights into parasite biology. Using a microfluidic platform, Lu et al.^96^ investigated the adhesion dynamics of *G. duodenalis*, a process central to the pathogenesis of giardiasis. In this model, controlled fluid flow through microchannels generated defined shear force gradients, allowing quantitative assessment of trophozoite adhesion strength. Similarly, Hansen and Fletcher^97^ employed a closed-flow chamber assay to evaluate the effects of rapid changes in osmolality, tonicity, and pH on *Giardia* adhesion to glass surfaces and intestinal cell monolayers. In another cell-free approach, Hochstetter et al.^98^ developed a microfluidic-based single-cell viability assay for *T. brucei*, combining chemical gradients with optical micromanipulation (Figure 6B). This platform enabled real-time monitoring of drug and chemical effects on parasite motility, allowing discrimination between cytocidal and cytostatic responses, optimization of effective dosages, and analysis of drug-induced alterations in cell motility.

Among OOC platforms, the intestinal chip is currently the most widely employed model for studying protozoan infections. One of the first studies to investigate parasitic infection using an intestinal OOC was conducted by Nikolaev et al.^99^ In this model, a central microchannel was coated with hydrogels and laser-sculpted to reproduce the geometry of intestinal crypts, followed by perfusion with LGR5-eGFP^+^ intestinal stem cells. The system included external media reservoirs and inlet-outlet channels that enabled controlled luminal flow and delivery of growth factors to the basal surface of the tissue. Using this platform, long-term infection by *C. parvum* was successfully modeled, with parasites completing their life cycle, including oocyst production, for more than 20 days without compromising tissue integrity.

Subsequent studies have further explored *Cryptosporidium* spp. infection using intestinal OOCs. Gunasekera et al.^100,101^ employed a pump-free, tubeless microfluidic device in which fluid shear stress was generated by evaporation-driven flow. Using the HCT-8 cell line to mimic the intestinal epithelium, these studies successfully reproduced the complete infection cycles of *C. parvum* ^100^ and *Cryptosporidium hominis*,^101^ reinforcing the suitability of OOC platforms for studying parasite development under physiologically relevant conditions.

Considering that the intestine is subjected to continuous mechanical stress due to peristalsis, Boquet-Pujadas et al.^102^ developed a mechanically active intestinal OOC model compatible with confocal microscopy. Infection experiments with *Entamoeba histolytica* revealed enhanced parasite virulence under peristaltic stimulation, as evidenced by increased host cell mortality, disruption of tissue junctions, degradation of actin in the epithelial brush border, phagocytosis of dead cells, and cleavage of E-cadherin. Additionally, increased amoebic penetration across the epithelium was observed, recapitulating ulcerative lesions characteristic of amoebiasis (Figure 6C).

Several OOC models have also been designed to investigate immune cell recruitment and activation across the intestinal barrier. The platform developed by Humayun et al.^103^ consisted of two hollow microtubes embedded within an ECM gel: one lined with endothelial cells to simulate blood vessels and perfused with immune cells such as neutrophils, and the other lined with intestinal epithelial cells. This configuration reproduced essential geometric and functional features of the gastrointestinal lumen and adjacent vasculature (Figure 6D). Upon infection with *T. gondii*, the system supported parasite replication and translocation across the epithelial barrier, resulting in increased epithelial permeability, while also enabling analysis of neutrophil migration and cytokine modulation associated with host immune responses. Similarly, Kim et al.^104^ employed a three-dimensional microfluidic assay simulating a microvasculature to reproduce the lytic cycle of *T. gondii*, allowing detailed investigation of both paracellular and transcellular migration of tachyzoites across biological barriers, including the intestinal, blood-brain, blood-ocular, and placental barriers.

A major advantage of OOC platforms lies in their capacity to integrate multiple tissues within a single microphysiological system. This feature is particularly relevant for complex infections such as malaria, in which the liver, spleen, and vascular system play central roles in disease pathogenesis. Rupar et al.^105,106^ developed a multiorgan OOC model integrating human liver, spleen, and endothelial tissues with *P. falciparum*-infected blood (Figure 6E). This system supported the survival of all intraerythrocytic stages of the parasite and enabled interorgan crosstalk, providing a robust preclinical framework for investigating malaria pathophysiology. Moreover, the platform allowed evaluation of antimalarial drugs, including chloroquine, lumefantrine, and artesunate, and enabled prediction of drug efficacy and toxicity in humans through in vitro pharmacokinetic and pharmacodynamic analyses.^106^

Microfluidic platforms have also been extensively used to dissect the biomechanical and cellular determinants of malaria severity. Alterations in the mechanical properties of infected erythrocytes, such as increased stiffness and capillary obstruction, are central to severe malaria manifestations. To characterize erythrocyte behavior under flow, Shelby et al.^107^ developed microfluidic channels of varying widths to analyze the passage of infected and uninfected red blood cells. This model revealed parasite stage-dependent cell deformation and recapitulated the splenic “pitting” process, whereby parasites are removed without destruction of erythrocytes. This phenomenon was further investigated using microfluidic devices mimicking interendothelial slits of the spleen, enabling precise control of mechanical stress through modulation of slit size and flow rate.^108^

In addition to biomechanical factors, interactions between host ligands and parasitized erythrocytes critically shape malaria outcomes. Antia et al.^109^ investigated these interactions using synthetic microfluidic channels resembling capillary networks and coated with purified host proteins or mammalian cells expressing host ligands. This approach enabled simultaneous modeling of infected erythrocyte adhesion under flow, channel size-dependent variations in adhesion, and macrophage-mediated phagocytosis in a physiologically relevant microvascular environment.

Placental malaria has likewise been investigated using microfluidic technologies. A placenta-on-a-chip model was developed to simulate the maternal–fetal interface by incorporating trophoblastic cells exposed to infected or uninfected maternal blood on one side of an ECM gel and human umbilical vein endothelial cells exposed to fetal blood on the opposite side. This configuration enabled the formation of a functional physiological barrier and facilitated the study of nutrient exchange and infection-driven alterations at the maternal–fetal interface.^110^

## 4 Technical Challenges and Future Perspectives

Despite the rapid development of advanced in vitro models, several technical and conceptual challenges still limit their widespread application in parasitology research (Table 2). One major hurdle is the intrinsic biological complexity of parasitic life cycles, which often involve multiple developmental stages, distinct host cell types, and dynamic transitions between tissues or even hosts. Reproducing these spatiotemporal dynamics in vitro remains difficult, particularly for parasites that require sequential cues from immune, stromal, and vascular compartments. Moreover, long-term maintenance of infections in complex systems is often constrained by limited nutrient diffusion, accumulation of waste products, and difficulties in controlling parasite burden without disrupting host tissue integrity. The fact that the life cycles of some parasites are being fully reproduced in advanced models, such as that of *Cryptosporidium* spp.,^99–101^ opens up possibilities for further research. Additionally, many advanced models rely on primary cells or stem cell-derived tissues, which can suffer from limited availability, donor-to-donor variability, high costs, and reduced reproducibility across laboratories. Standardization of protocols, readouts, and validation criteria is therefore a critical unmet need.

**Table 2:**
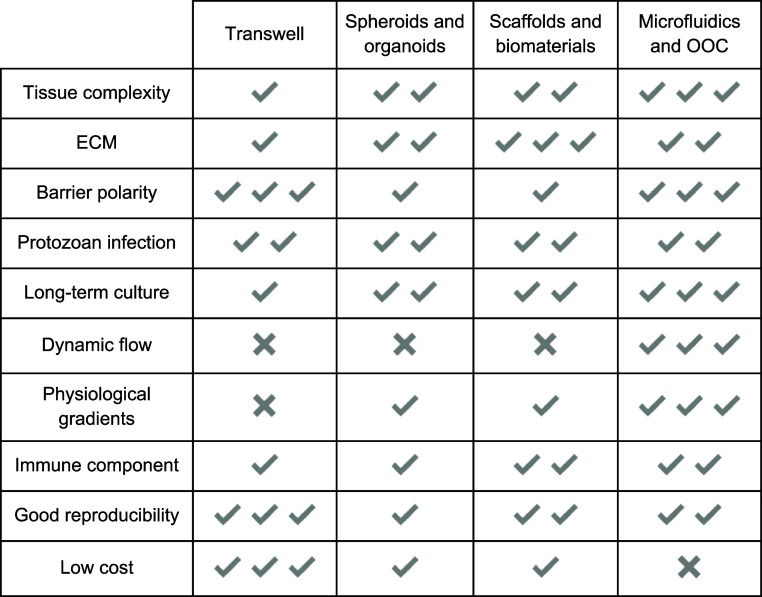
Advantages and Limitations of Advanced In Vitro Culture Models

From a technical standpoint, integrating immune components into advanced culture systems remains especially challenging, yet essential for studying host–parasite interactions. Most current models lack functional innate and adaptive immune responses, which play decisive roles in parasite invasion, persistence, and pathology. Analytical limitations also persist: high-resolution imaging, real-time monitoring, and quantitative readouts compatible with 3D or microfluidic platforms are still less accessible than conventional assays used in 2D cultures. Research has been refining techniques applied to complex models, for example, with the application of 3D microscopy videos to a mechanically active and deformable OOC intestinal, simulating peristalsis.^102^

Looking forward, future perspectives point toward increasing model integration and technological convergence. The combination of organoids with microfluidics, biosensors, and advanced imaging approaches is expected to enable more physiologically relevant and dynamic representations of parasitic infections. Incorporation of immune cells, microbiota components, and vascular-like networks will further enhance model fidelity. In parallel, advances in bioengineering, automation, and artificial intelligence-based image and data analysis may improve scalability, reproducibility, and throughput, facilitating drug screening and mechanistic studies.^116–118^ Ultimately, while advanced in vitro models will not fully replace in vivo systems in the near future, their continued refinement positions them as powerful complementary tools to bridge the gap between simplified cell cultures and complex animal models in parasitology research.

## 5 Conclusions

Advanced in vitro models provide more physiologically relevant platforms to study host–parasite interactions than conventional two-dimensional cultures, enabling improved mechanistic and translational insights. Although challenges related to complexity, standardization, and immune integration remain, ongoing technological advances are steadily enhancing their robustness and applicability. As complementary tools to in vivo models, these systems are expected to play an increasingly important role in parasitology research and antiparasitic drug development.

## Supplementary Material



## Notes

Table S1 (Overview of advanced in vitro models applied to protozoan infections) (PDF)

## List of Abbreviations

ARPE-19: human retinal pigment epithelial cells
BeWo: human trophoblast cell line
BV2: murine microglial cell line
Caco-2: human colorectal adenocarcinoma cells
COS-7: fibroblast-like cell line from African green monkey kidney
CHO: chinese hamster ovary cells
FC: flow cytometry
HBMECs: human brain microvascular endothelial cells
HCT-8: human ileocecal colorectal adenocarcinoma cells
HeLa: human cervical cancer cells
HFF: human foreskin fibroblast cells
HIE: human intestinal enteroids
HT22: murine hippocampal neuronal cell line
HUVEC: human umbilical vein endothelial cells
hFLOs: human fetal liver organoids
hMVECs: human microvascular endothelial cells
ICC: immunocytochemistry
IHC: immunohistochemistry
IFI: indirect immunofluorescence
iPSCs: induced pluripotent stem cells
ISC: intestinal stem Cell
JEG-3: human trophoblast carcinoma-derived cells
LGR5-eGFP+: intestinal stem cells
LLC-MK2: cell line used as a source of *Trypanosoma cruzi*
MDCK: Madin–Darby canine kidney epithelial cell line
MIE: murine intestinal enteroids
NKE: normal kidney epithelial cell lines
ODMs: organoid-derived monolayers
PCR: polymerase chain reaction
RAW: murine monocyte/macrophage-like cell line
RBC: red blood cell
RIT: mouse rectal tumor cells
scRNA-seq: single-cell RNA sequencing
SEM: scanning electron microscopy
SHG: second harmonic generation microscopy
TEM: transmission electron microscopy
TEER: transepithelial/transendothelial electrical resistance
THP-1: human monocytic cell line
Vero: continuous epithelial cell line from African green monkey
WB: western blotting

## References

[1] Horan, N. P. Handbook of Water and Wastewater Microbiology; Mara, D. , Horan, N. , Eds.; Academic Press: London, 2003.

[2] Wang Y., Liang J., Yang C., Wang J., Zhu Q., Tong G., Li T., Li G., Huang Y., Yang Y., Ren J., Li Y., Shen Y., Zhao Y. (2025). CXCL10̂high TNFα̂high Ki67̂+ Microglia Recruit
and Activate CD8̂+ T Cells in the Brainstem during Experimental
Cerebral Malaria. CNS Neurosci. Ther..

[3] Suleiman J. B., Azlan M. (2025). Burden and Distribution of Protozoan Pathogens in Diarrhea Cases
Worldwide: A Systematic Review and Meta-Analysis, 1999–2024. Cureus.

[4] Hagemann C. L., Kist T. B. L., da Silva P. E. A. (2023). Mecanismos Celulares de Patogenicidade
em Protozoários de Importância Médica. Rev. Liberato.

[5] Baker B. M., Chen C. S. (2012). Deconstructing the
Third Dimension: How 3D Culture
Microenvironments Alter Cellular Cues. J. Cell
Sci..

[6] Duval K., Grover H., Han L.-H., Mou Y., Pegoraro A. F., Fredberg J., Chen Z. (2017). Modeling Physiological
Events in
2D vs. 3D Cell Culture. Physiology.

[7] World Health Organization . Ending the Neglect to Attain the Sustainable Development Goals: A Road Map for Neglected Tropical Diseases 2021–2030; World Health Organization: Geneva, 2020.

[8] Langhans S. A. (2018). Three-Dimensional
in Vitro Cell Culture Models in Drug Discovery and Drug Repositioning. Front. Pharmacol..

[9] Danielson J. J., Perez N., Romano J. D., Coppens I. (2018). Modelling *Toxoplasma
gondii* Infection in a 3D Cell Culture System in Vitro: Comparison
with Infection in 2D Cell Monolayers. PLoS One.

[10] Silberstein E., Kim K. S., Acosta D., Debrabant A. (2021). Human Placental
Trophoblasts Are Resistant to *Trypanosoma cruzi* Infection
in a 3D-Culture Model. Front. Microbiol..

[11] O’Keeffe A., Hale C., Cotton J. A., Yardley V., Gupta K., Ananthanarayanan A., Murdan S., Croft S. L. (2020). Novel 2D
and 3D
Assays to Determine the Activity of Anti-Leishmanial Drugs. Microorganisms.

[12] Orlando L. M. R., Lara L. d. S., de
Souza T. P., Paes V. B., Calvet C. M., de Mesquita L. B., Lechuga G. C., Pereira C. N., dos Santos M. S., Pereira M. C. d. S. (2025). Pyrazole-Imidazoline Derivative Prevents Cardiac Damage
and Mortality in Acute *Trypanosoma cruzi* Infection. Pharmaceuticals.

[13] Boström S., Schmiegelow C., Abu Abed U., Minja D. T. R., Lusingu J., Brinkmann V., Honkpehedji Y. J., Loembe M. M., Adegnika A. A., Mordmüller B., Troye-Blomberg M., Amulic B. (2017). Neutrophil Alterations
in Pregnancy-Associated Malaria and Induction of Neutrophil Chemotaxis
by *Plasmodium falciparum*. Parasite
Immunol..

[14] Justus C. R., Marie M. A., Sanderlin E. J., Yang L. V. (2014). Transwell *in Vitro* Cell Migration
and Invasion Assays. Methods Mol. Biol..

[15] Hoffmann P., Burmester M., Langeheine M., Brehm R., Empl M. T., Seeger B., Breves G. (2021). Caco-2/HT29-MTX Co-Cultured Cells
as a Model for Studying Physiological Properties and Toxin-Induced
Effects on Intestinal Cells. PLoS One.

[16] Jung S. M., Kim S. (2022). In Vitro Models of
the Small Intestine for Studying Intestinal Diseases. Front. Microbiol..

[17] Nash T. E. (2019). Long-Term
Culture of *Giardia lamblia* in Cell Culture Medium
Requires Association with Viable Mammalian Cells. Infect. Immun..

[18] Rigamonti G., Veronesi F., Chiaradia E., Gosten-Heinrich P., Müller A., Brustenga L., de Angelis S., Tognoloni A., De Santo R., Klotz C., Lalle M. (2025). Selective
Activity of Tabebuia avellanedae against *Giardia duodenalis* infecting organoid-derived human gastrointestinal epithelia. Int. J. Parasitol. Drugs Drug Resist..

[19] Fisher B. S., Estraño C. E., Cole J. A. (2013). Modeling Long-Term Host Cell-*Giardia lamblia* Interactions. PLoS
One.

[20] Holthaus D., Delgado-Betancourt E., Aebischer T., Seeber F., Klotz C. (2021). Harmonization
of Protocols for Multi-Species Organoid Platforms to Study Intestinal
Biology of *Toxoplasma gondii*. Front. Cell. Infect. Microbiol..

[21] Holthaus D., Kraft M. R., Krug S. M., Wolf S., Müller A., Delgado Betancourt E., Schorr M., Holland G., Knauf F., Schulzke J. D. (2022). Dissection of Barrier Dysfunction in Organoid-Derived
Human Intestinal Epithelia Induced by *Giardia duodenalis*. Gastroenterology.

[22] Kraft M. R., Klotz C., Bücker R., Schulzke J. D., Aebischer T. (2017). Giardia’s
Epithelial Cell Interaction *in Vitro*. Front. Cell. Infect. Microbiol..

[23] Kumar A., Chatterjee I., Anbazhagan A. N., Jayawardena D., Priyamvada S., Alrefai W. A., Sun J., Borthakur A., Dudeja P. K. (2018). *Cryptosporidium parvum* Disrupts Intestinal
Epithelial Barrier Function via Altering Expression of Key Tight Junction
and Adherens Junction Proteins. Cell. Microbiol..

[24] Hernández M., Wicz S., Corral R. S. (2016). Cardioprotective Actions of Curcumin
during *Trypanosoma cruzi* Infection. Phytomedicine.

[25] Maeda F. Y., Alves R. M., Cortez C., Lima F. M., Yoshida N. (2016). Host Cell
Invasion and Oral Infection by *Trypanosoma cruzi*. Parasit. Vectors.

[26] Tao Q., Yang D., Qin K., Liu L., Jin M., Zhang F., Zhu J., Wang J., Luo Q., Du J., Yu L., Shen J., Chu D. (2023). Studies on
the Mechanism
of *Toxoplasma gondii* Chinese 1 Genotype Wh6 Strain
Causing Mice Abnormal Cognitive Behavior. Parasit.
Vectors.

[27] Song H. B., Jun H.-O., Kim J. H., Lee Y.-H., Choi M.-H., Kim J. H. (2017). Disruption of Outer
Blood-Retinal Barrier by *Toxoplasma gondii*-Infected
Monocytes Is Mediated by Paracrinely
Activated FAK Signaling. PLoS One.

[28] Ashander L. M., Lie S., Ma Y., Rochet E., Washington J. M., Furtado J. M., Appukuttan B., Smith J. R. (2019). Neutrophil Activities
in Human Ocular Toxoplasmosis: An *in Vitro* Study
with Human Cells. Invest. Ophthalmol. Vis. Sci..

[29] Jiang D., Wu S., Xu L., Xie G., Li D., Peng H. (2022). Anti-Infection
Roles of miR-155–5p Packaged in Exosomes Secreted by Dendritic
Cells Infected with *Toxoplasma gondii*. Parasit. Vectors.

[30] Reddy S. B., Nagy N., Rönnberg C., Chiodi F., Lugaajju A., Heuts F., Szekely L., Wahlgren M., Persson K. E. M. (2021). Direct
contact between *Plasmodium falciparum* and human B-cells
in a novel co-culture increases parasite growth and affects B-cell
growth. Malar. J..

[31] Yeste J., Illa X., Alvarez M., Villa R. (2018). Engineering and Monitoring
Cellular Barrier Models. J. Biol. Eng..

[32] Spring K. R. (1998). Routes
and Mechanism of Fluid Transport by Epithelia. Annu. Rev. Physiol..

[33] Srinivasan B., Kolli A. R., Esch M. B., Abaci H. E., Shuler M. L., Hickman J. J. (2015). TEER Measurement
Techniques for *in Vitro* Barrier Model Systems. J. Lab. Autom..

[34] Zucco F., Batto A. F., Bises G., Chambaz J., Chiusolo A., Consalvo R., Cross H., Dal Negro G., de Angelis I., Fabre G., Guillou F., Hoffman S., Laplanche L., Morel E., Pinçon-Raymond M., Prieto P., Turco L., Ranaldi G., Rousset M., Sambuy Y., Scarino M. L., Torreilles F., Stammati A. (2005). An inter-laboratory study to evaluate the effects of
medium composition on the differentiation and barrier function of
Caco-2 cell lines. Altern. Lab. Anim..

[35] Galipeau H. J., Verdu E. F. (2016). Measuring Intestinal
Permeability Using Functional
Assays. Neurogastroenterol. Motil..

[36] González-González M., Díaz-Zepeda C., Eyzaguirre-Velásquez J., González-Arancibia C., Bravo J. A., Julio-Pieper M. (2019). Investigating
Gut Permeability in Animal Models of Disease. Front. Physiol..

[37] González-Mariscal L., Betanzos A., Nava P., Jaramillo B. E. (2003). Tight Junction
Proteins. Prog. Biophys. Mol. Biol..

[38] Anderson J. M., Van Itallie C. M. (2009). Physiology and Function of the Tight
Junction. Cold Spring Harb. Perspect. Biol..

[39] Kitel R., Czarnecka J., Rusin A. (2013). Three-Dimensional Cell
Cultures:
Applications in Basic Science and Biotechnology. Postepy Biochem..

[40] Białkowska K., Komorowski P., Bryszewska M., Miłowska K. (2020). Spheroids
as a Type of Three-Dimensional Cell Cultures—Examples of Methods
of Preparation and the Most Important Applications. Int. J. Mol. Sci..

[41] Sant S., Johnston P. A. (2017). Production of 3D
Tumor Spheroids. Drug Discovery Today Technol..

[42] Sutherland, R. M. ; Durand, R. E. Growth and Cellular Characteristics of Multicell Spheroids. In Recent Results in Cancer Research; Springer: Berlin, 1984; Vol. 95, pp 24–49. 6396760 10.1007/978-3-642-82340-4_2

[43] Živković Z., Opačak-Bernardi T. (2025). Overview on Spheroid and Organoid
Models. Sci..

[44] Chua A. C. Y., Ananthanarayanan A., Ong J. J. Y., Wong J. Y., Yip A., Singh N. H., Qu Y., Dembele L., McMillian M., Ubalee R., Davidson S., Tungtaeng A., Imerbsin R., Gupta K., Andolina C., Lee F., S-W Tan K., Nosten F., Russell B., Lange A., Diagana T. T., Rénia L., Yeung B. K. S., Yu H., Bifani P. (2019). Hepatic Spheroids Used as an *in Vitro* Model to Study Malaria Relapse. Biomaterials.

[45] Ferrão M. P., Nisimura M. L., Moreira O. C., Land M. G., Pereira M. C., de Mendonça-Lima L., Araujo-Jorge T. C., Waghabi M. C., Garzoni L. R. (2018). Inhibition of TGF-β
Pathway
Reverts Extracellular Matrix Remodeling in *Trypanosoma cruzi*-Infected Cardiac Spheroids. Exp. Cell Res..

[46] Rodríguez M. E., Rizzi M., Caeiro L., Masip Y., Sánchez D. O., Tekiel V. (2019). Transmigration of *Trypanosoma cruzi* Trypomastigotes through 3D Spheroids Mimicking
Host Tissues. Methods Mol. Biol..

[47] Silberstein E., Kim K. S., Acosta D., Debrabant A. (2021). Human Placental
Trophoblasts Are Resistant to *Trypanosoma cruzi* Infection
in a 3D-Culture Model of the Maternal-Fetal Interface. Front. Microbiol..

[48] de
Almeida Fiuza L. F., Batista D. D. G. J., Nunes D. F., Moreira O. C., Cascabulho C., Soeiro M. N. C. (2021). Benznidazole Modulates Release of
Inflammatory Mediators by Cardiac Spheroids Infected with *Trypanosoma cruzi*. Exp. Parasitol..

[49] Garzoni L. R., Adesse D., Soares M. J., Rossi M. I. D., Borojevic R., Meirelles M. (2008). Fibrosis and Hypertrophy Induced
by *Trypanosoma
cruzi* in a Three-Dimensional Cardiomyocyte-Culture System. J. Infect. Dis..

[50] Correa
Leite P. E., de Araujo Portes J., Pereira M. R., Russo F. B., Martins-Duarte E. S., Almeida Dos Santos N., Attias M., Barrantes F. J., Baleeiro Beltrão-Braga P. C., de Souza W. (2021). Morphological and Biochemical
Repercussions of *Toxoplasma gondii* Infection in a
3D Human Brain Neurospheres Model. Brain Behav.
Immun. Health.

[51] Yang A. S. P., Dutta D., Kretzschmar K., Hendriks D., Puschhof J., Hu H., Boonekamp K. E., van Waardenburg Y., Chuva de Sousa Lopes S. M., van Gemert G. J., de Wilt J. H. W., Bousema T., Clevers H., Sauerwein R. W. (2023). Development
of *Plasmodium falciparum* Liver Stages in Hepatocytes
Derived from Human Fetal Liver Organoid
Cultures. Nat. Commun..

[52] Yan H. H. N., Chan A. S., Lai F. P., Leung S. Y. (2023). Organoid Cultures
for Cancer Modeling. Cell Stem Cell.

[53] Seo H. H., Han H. W., Lee S. E., Hong S. H., Cho S. H., Kim S. C., Koo S. K., Kim J. H. (2020). Modelling *Toxoplasma gondii* Infection in Human Cerebral Organoids. Emerg. Microbes Infect..

[54] Chandrasegaran P., Nabilla Lestari A., Sinton M. C., Gopalakrishnan J., Quintana J. F. (2023). Modelling Host-*Trypanosoma brucei gambiense* Interactions *in Vitro* Using Human Induced Pluripotent
Stem Cell-Derived Cortical Brain Organoids. F1000Res..

[55] Martorelli
Di Genova B., Wilson S. K., Dubey J. P., Knoll L. J. (2019). Intestinal
Delta-6-Desaturase Activity Determines Host Range for Toxoplasma Sexual
Reproduction. PLoS Biol..

[56] Zhang X. T., Gong A. Y., Wang Y., Chen X., Lim S. S., Dolata C. E., Chen X. M. (2016). *Cryptosporidium
parvum* Infection Attenuates the Ex Vivo Propagation of Murine
Intestinal
Enteroids. Physiol. Rep..

[57] Heo I., Dutta D., Schaefer D. A., Iakobachvili N., Artegiani B., Sachs N., Boonekamp K. E., Bowden G., Hendrickx A. P. A., Willems R. J. L., Peters P. J., Riggs M. W., O’Connor R., Clevers H. (2018). Modelling Cryptosporidium
Infection in Human Small Intestinal and Lung Organoids. Nat. Microbiol..

[58] Bhalchandra S., Lamisere H., Ward H. (2020). Intestinal
Organoid/Enteroid-Based
Models for Cryptosporidium. Curr. Opin. Microbiol..

[59] Cancela S., Sena F., Pagotto R., Crispo M., Francia M. E., Bollati-Fogolín M. (2025). Enhancing
Pre-Sexual and Sexual Differentiation
of *Toxoplasma gondii* Using Retinal Epithelial Cells
and Intestinal Organoids. Cell Rep..

[60] Mellin R., Boddey J. A. (2020). Organoids for Liver-Stage Malaria
Research. Trends Parasitol..

[61] Park S. Y., Hong H. J., Lee H. J. (2023). Fabrication of Cell
Spheroids for
3D Cell Culture and Biomedical Applications. BioChip J..

[62] Zhao Z., Chen X., Dowbaj A. M., Sljukic A., Bratlie K., Lin L., Fong E. L. S., Balachander G. M., Chen Z., Soragni A., Huch M., Zeng Y. A., Wang Q., Yu H. (2022). Organoids. Nat. Rev. Methods Primers.

[63] Shimojo, A. ; Rodrigues, I. ; Perez, A. ; Souto, E. B. ; Pellizzer Gabriel, L. ; Webster, T. Scaffolds for Tissue Engineering: A State-of-the-Art Review Concerning Types, Properties, Materials, Processing, and Characterization. In Racing for the Surface: Antimicrobial and Interface Tissue Engineering; Springer: Cham, 2020; pp 647–676.

[64] Gervaso F., Sannino A., Peretti G. M. (2013). The Biomaterialist’s
Task:
Scaffold Biomaterials and Fabrication Technologies. Joints.

[65] Dhandayuthapani B., Yoshida Y., Maekawa T., Kumar D. S. (2011). Polymeric Scaffolds
in Tissue Engineering Application: A Review. Int. J. Polym. Sci..

[66] Roshandel M., Dorkoosh F. (2021). Cardiac Tissue Engineering,
Biomaterial Scaffolds,
and Their Fabrication Techniques. Polym. Adv.
Technol..

[67] Ghalsasi P., Chithiravelu G., Joddar B. (2025). Seaweed-Derived Polysaccharides as
Sustainable Biomaterials for Tissue Engineering Applications. ACS Biomater. Sci. Eng..

[68] Tajurahim N. A. N., Mahmood S., Ngadiman N. H. A., Sing S. L. (2025). Biomaterials for
Tissue Engineering Scaffolds: Balancing Efficiency and Eco-Friendliness
through Life Cycle Assessment. Cleaner Environ.
Syst..

[69] Deb P., Deoghare A. B., Borah A., Barua E., Das Lala S. (2018). Scaffold Development
Using Biomaterials. Mater. Today Proc..

[70] Mahmoud E. M., Sayed M., Mansour T. S., Naga S. M. (2025). Biodegradable Ceramic
Materials for Orthopedic and Dental Applications. Discover Appl. Sci..

[71] Park S. M., Ryoo J. H., Kwon H. C., Han S. G. (2025). Scaffold Biomaterials
in the Development of Cultured Meat: A Review. Food Sci. Anim. Resour..

[72] Jang J., Min K., Kim C., Shin J., Lee J., Yi S. (2023). Scaffold Characteristics,
Fabrication Methods, and Biomaterials for Bone Tissue Engineering. Int. J. Precis. Eng. Manuf..

[73] Khan A. R., Gholap A. D., Grewal N. S., Jun Z., Khalid M., Zhang H. J. (2025). Advances in Smart Hybrid Scaffolds
for Regenerative
Clinical Applications. Eng. Regen..

[74] Sindhi K., Pingili R. B., Beldar V., Bhattacharya S., Rahaman J., Mukherjee D. (2025). The Role of Biomaterials-Based Scaffolds
in Advancing Skin Tissue Constructs. J. Tissue
Viability.

[75] O’Brien F. J. (2011). Biomaterials
and Scaffolds for Tissue Engineering. Mater.
Today.

[76] Wu S., Qin F., Meng Y. (2025). Antimicrobial
Drug-Loaded Scaffold Materials for Treatment
of Bone Infections. Nanoscale Horiz..

[77] Picado-Tejero D., Mendoza-Cerezo L., Rodríguez-Rego J. M., Carrasco-Amador J. P., Marcos-Romero A. C. (2025). Recent Advances in 3D Bioprinting of Porous Scaffolds
for Tissue Engineering. J. Funct. Biomater..

[78] Velasco M. A., Narváez-Tovar C. A., Garzón-Alvarado D. A. (2015). Design,
Materials, and Mechanobiology of Biodegradable Scaffolds for Bone
Tissue Engineering. BioMed. Res. Int..

[79] Tabaei S. J. S., Rahimi M., Akbaribazm M., Ziai S. A., Sadri M., Shahrokhi S. R., Rezaei M. S. (2020). Chitosan-Based Nano-Scaffolds as
Antileishmanial Wound Dressing. Iran. J. Basic
Med. Sci..

[80] Bhattacharya S., Bhattacharyya T., Khanra S., Banerjee R., Dash J. (2023). Nucleoside-Derived
Metallohydrogel Induces Cell Death in Leishmania Parasites. ACS Infect. Dis..

[81] Gutekunst S. B., Siemsen K., Huth S., Möhring A., Hesseler B., Timmermann M., Paulowicz I., Mishra Y. K., Siebert L., Adelung R. (2019). 3D Hydrogels
Containing Interconnected Microchannels for Capturing Pathogenic *Acanthamoeba castellanii*. ACS Biomater.
Sci. Eng..

[82] Wang J., Song L., Xing Y., Dai Y., Hu J., Qu G., Xu Y., Yin X., Hang D., Zhang J. (2025). Sustained-Release
Collagen Hydrogels for Prevention and Treatment of Schistosomiasis. Microbiol. Spectr..

[83] Feng Y., Wang F., Zhang X. W., Bhutani H., Ye B. (2017). Characterizations
and Bioactivities of Albendazole Sulfoxide-Loaded Thermo-Sensitive
Hydrogel. Parasitol. Res..

[84] Zubiría I., Abreu I., Boso D., Pérez G., Cazapal C., Sánchez-Andrade R., Arias M. S., Paz-Silva A., Hernández J. A., Camiña M. (2025). Agar-Agar
Gels Carrying Curative and Preventive Agents against Helminths. Gels.

[85] Murphy S. V., Atala A. (2014). 3D Bioprinting of Tissues and Organs. Nat.
Biotechnol..

[86] Heinrich M. A., Liu W., Jimenez A., Yang J., Akpek A., Liu X., Pi Q., Mu X., Hu N., Schiffelers R. M., Prakash J., Xie J., Zhang Y. S. (2019). 3D Bioprinting:
From Benches to Translational Applications. Small.

[87] Iyer K. S., Bao L., Zhai J., Jayachandran A., Luwor R., Li J. J., Li H. (2025). Microgel-Based Bioink
for Extrusion-Based 3D Bioprinting. Bioact.
Mater..

[88] Zhang Y. S., Yue K., Aleman J., Mollazadeh-Moghaddam K., Bakht S. M., Yang J., Jia W., Dell’Erba V., Assawes P., Shin S. R., Dokmeci M. R., Oklu R., Khademhosseini A. (2017). 3D Bioprinting for Tissue and Organ
Fabrication. Ann. Biomed. Eng..

[89] Li W. J., Laurencin C. T., Caterson E. J., Tuan R. S., Ko F. K. (2002). Electrospun
Nanofibrous Structure: A Novel Scaffold for Tissue Engineering. J. Biomed. Mater. Res..

[90] Tanzli E., Ehrmann A. (2021). Electrospun Nanofibrous
Membranes for Tissue Engineering
and Cell Growth. Appl. Sci..

[91] Doganay M. T., Chelliah C. J., Tozluyurt A., Hujer A. M., Obaro S. K., Gurkan U., Patel R., Bonomo R. A., Draz M. (2023). 3D Printed
Materials for Combating Antimicrobial Resistance. Mater. Today.

[92] Regmi S., Poudel C., Adhikari R., Luo K. Q. (2022). Applications of
Microfluidics and Organ-on-a-Chip in Cancer Research. Biosensors.

[93] Low L. A., Mummery C., Berridge B. R., Austin C. P., Tagle D. A. (2021). Organs-on-Chips:
Into the Next Decade. Nat. Rev. Drug Discovery.

[94] Sadeghzade S., Hooshiar M. H., Akbari H., Tajer M. H. M., Sahneh K. K., Ziaei S. Y., Jalali F., Akouchakian E. (2024). Recent Advances
in Organ-on-a-Chip Models: How Precision Engineering Integrates Cutting-Edge
Technologies in Fabrication and Characterization. Appl. Mater. Today.

[95] Vunjak-Novakovic G., Ronaldson-Bouchard K., Radisic M. (2021). Organs-on-Chips Models for Biological
Research. Cell.

[96] Lu L., Zheng G. X., Yang Y. S., Feng C. Y., Liu F. F., Wang Y. H. (2017). Measurement of *Giardia lamblia* Adhesion
Force Using an Integrated Microfluidic Assay. Anal. Bioanal. Chem..

[97] Hansen W. R., Fletcher D. A. (2008). Tonic Shock Induces Detachment of *Giardia lamblia*. PLoS Negl. Trop. Dis..

[98] Hochstetter A., Stellamanns E., Deshpande S., Uppaluri S., Engstler M., Pfohl T. (2015). Microfluidics-Based
Single Cell Analysis Reveals Drug-Dependent Motility
Changes in Trypanosomes. Lab Chip.

[99] Nikolaev M., Mitrofanova O., Broguiere N., Geraldo S., Dutta D., Tabata Y., Elci B., Brandenberg N., Kolotuev I., Gjorevski N., Clevers H., Lutolf M. P. (2020). Homeostatic
Mini-Intestines through Scaffold-Guided Organoid Morphogenesis. Nature.

[100] Gunasekera S., Thierry B., Cheah E., King B., Monis P., Carr J. M., Chopra A., Watson M., O’Dea M., Ryan U. (2024). A Pumpless and Tubeless
Microfluidic
Device Enables Extended *in Vitro* Development of *Cryptosporidium parvum*. Open Forum
Infect. Dis..

[101] Gunasekera S., Thierry B., King B., Monis P., Carr J. M., Chopra A., Watson M., O’Dea M., Cheah E., Ram R., Clode P. L., Hijjawi N., Ryan U. (2025). Microphysiological Gut-on-Chip Enables Extended *in Vitro* Development of *Cryptosporidium hominis*. Front. Cell. Infect. Microbiol..

[102] Boquet-Pujadas A., Feaugas T., Petracchini A., Grassart A., Mary H., Manich M., Gobaa S., Olivo-Marin J. C., Sauvonnet N., Labruyère E. (2022). 4D Live Imaging
and Computational Modeling of a Functional Gut-on-a-Chip Evaluate
How Peristalsis Facilitates Enteric Pathogen Invasion. Sci. Adv..

[103] Humayun M., Ayuso J. M., Park K. Y., Martorelli
Di Genova B., Skala M. C., Kerr S. C., Knoll L. J., Beebe D. J. (2022). Innate Immune Cell Response to Host-Parasite Interaction
in a Human Intestinal Tissue Microphysiological System. Sci. Adv..

[104] Kim H., Hong S. H., Jeong H. E., Han S., Ahn J., Kim J. A., Yang J. H., Oh H. J., Chung S., Lee S. E. (2022). Microfluidic Model for *in Vitro* Acute *Toxoplasma gondii* Infection and Transendothelial Migration. Sci. Rep..

[105] Rupar M. J., Sasserath T., Smith E., Comiter B., Sriram N., Long C. J., McAleer C. W., Hickman J. J. (2023). Development
of a Human Malaria-on-a-Chip Disease Model for Drug Efficacy and Off-Target
Toxicity Evaluation. Sci. Rep..

[106] Rupar M. J., Hanson H. M., Botlick B. L., Sriram N., Rogers S., Zuniga J., Liu Z., Trimmer S. J., Ciurca J. M., Long C. J., McAleer C. W., Schmidt S., Favuzza P., Lowe P., Gobeau N., Hickman J. J. (2025). Translation
of a Human-Based Malaria-on-a-Chip Phenotypic Disease Model for in
Vivo Applications. Adv. Sci..

[107] Shelby J. P., White J., Ganesan K., Rathod P. K., Chiu D. T. (2003). A Microfluidic Model for Single-Cell Capillary Obstruction
by *Plasmodium falciparum*-Infected Erythrocytes. Proc. Natl. Acad. Sci. U.S.A..

[108] Elizalde-Torrent A., Trejo-Soto C., Méndez-Mora L., Nicolau M., Ezama O., Gualdrón-López M., Fernández-Becerra C., Alarcón T., Hernández-Machado A., Del Portillo H. A. (2021). Pitting
of Malaria Parasites in Microfluidic Devices Mimicking Spleen Interendothelial
Slits. Sci. Rep..

[109] Antia M., Herricks T., Rathod P. K. (2007). Microfluidic
Modeling
of Cell-Cell Interactions in Malaria Pathogenesis. PLoS Pathog..

[110] Mosavati B., Oleinikov A., Du E. (2022). 3D Microfluidics-Assisted
Modeling of Glucose Transport in Placental Malaria. Sci. Rep..

[111] Nahak B. K., Mishra A., Preetam S., Tiwari A. (2022). Advances in
Organ-on-a-Chip Materials and Devices. ACS Appl.
Bio Mater..

[112] Tajeddin A., Mustafaoglu N. (2021). Design and
Fabrication of Organ-on-Chips:
Promises and Challenges. Micromachines.

[113] Seiler S. T., Mantalas G. L., Selberg J., Cordero S., Torres-Montoya S., Baudin P. V., Ly V. T., Amend F., Tran L., Hoffman R. N., Rolandi M., Green R. E., Haussler D., Salama S. R., Teodorescu M. (2022). Modular Automated
Microfluidic Cell Culture Platform Reduces Glycolytic Stress in Cerebral
Cortex Organoids. Sci. Rep..

[114] Li Z., Zhao Y., Lv X., Deng Y. (2023). Integrated Brain-on-a-Chip
and Automated Organ-on-Chips Systems. Interdiscip.
Med..

[115] Morales I. A., Boghdady C. M., Campbell B. E., Moraes C. (2022). Integrating
Mechanical Sensor Readouts into Organ-on-a-Chip Platforms. Front. Bioeng. Biotechnol..

[116] Grexa I., Diosdi A., Harmati M., Kriston A., Moshkov N., Buzas K., Pietiäinen V., Koos K., Horvath P. (2021). SpheroidPicker for Automated 3D Cell
Culture Manipulation Using Deep Learning. Sci.
Rep..

[117] Dai Y., Wang P., Mishra A., You K., Zong Y., Lu W. F., Chow E. K., Preshaw P. M., Huang D., Chew J. R. J., Ho D., Sriram G. (2025). 3D Bioprinting
and
Artificial Intelligence-Assisted Biofabrication of Personalized Oral
Soft Tissue Constructs. Adv. Healthcare Mater..

[118] Zieger V., Frejek D., Zimmermann S., Miotto G. A. A., Koltay P., Zengerle R., Kartmann S. (2024). Automation
in 3D Cell Culture: High-Throughput Handling of Spheroids and Organoids. Adv. Healthcare Mater..

