# Supplementary material for: The Future of Protozoan Infection Research: 3D Cell Culture and beyond
Source: ACS Infect Dis. 2026 May 26;12(6):1852–65. doi: 10.1021/acsinfecdis.6c00244 (PMC13270520; doi:10.1021/acsinfecdis.6c00244)
Supplement: Supplementary file 1 [file id6c00244_si_001.pdf]

Supporting Information

The future of protozoan infection research: 3D cell culture and beyond

Sarah Beatriz de Fucio[1], Abel Sana[1], Beatriz Marques[3], Fernanda Araujo[1], Nusrat Sattar[1], Julia Charleaux[2], Jameel Inal[4,5], Marcel Ramirez[6], Izadora Volpato Rossi[7]*

1. Postgraduate Program in Cellular and Molecular Biology, Federal University of Paraná, Curitiba, Brazil
2. Postgraduate Program in Microbiology, Parasitology and Pathology, Federal University of Paraná, Curitiba, Brazil
3. Postgraduate Program in Biosciences and Biotechnology, Carlos Chagas Institute, Fundação Oswaldo Cruz, (FIOCRUZ-PR), Curitiba, Brazil
4. School of Human Sciences, Cell Communication in Disease Pathology, London Metropolitan University, London, UK
5. School of Life and Medical Sciences, Biosciences Research Group, University of Hertfordshire, Hatfield, UK
6. Carlos Chagas Institute, Fundação Oswaldo Cruz, (FIOCRUZ-PR), Curitiba, Brazil
7. Department of Immunology, Parasitology and Pathology, State University of Londrina, Londrina, Brazil

*Correspondences to: izadorarossi@uel.br or izadoravolpato@gmail.com

Table S1. Overview of advanced *in vitro* models applied to protozoan infections

Advanced *in vitro* model	Protozoan	Cell lineage(s)	Tissue/organ represented	Techniques in advanced infection study	Reference
Transwell	*Giardia duodenalis*	Caco-2	Intestine	TEER; ICC; analysis of Chemokine/Cytokine	Kraft et al., 2017
Transwell	*Giardia duodenalis*	Caco-2; RIT; Cos7	Intestine	Growth analysis of co-cultures	Nash, 2019
Transwell	*Giardia duodenalis*	Caco-2; MDCK	Intestine	ICC; TEER; cytotoxicity assays	Rigamonti et al., 2025
Transwell	*Giardia duodenalis*	ODMs	Intestine	TEER; permeability assay; ICC; transcriptomics; TEM; PCR; WB	Holthaus et al., 2022
Transwell	*Giardia duodenalis; Toxoplasma gondii*	ODMs	Intestine	RT-qPCR; TEER; ICC	Holthaus et al., 2021
Transwell	*Toxoplasma gondii*	THP-1; ARPE-19	Blood-retinal barrier	Analysis of Chemokine/Cytokine; TEER; WB; ICC	Song et al., 2017
Transwell	*Toxoplasma gondii*	BV2; HT22	Human brain	ICC; WB; RT-qPCR; FC	Tao et al., 2023
Transwell	*Toxoplasma gondii*	HFF; RAW	Blood	FC; WB; RT-qPCR	Jiang et al., 2022
Transwell	*Toxoplasma gondii*	Retinal epithelial cell; ARPE-19; neutrophils	Retina	Migration assay; RT-qPCR; analysis of Chemokine/Cytokine; Detection of ROS	Ashander et al., 2019
Transwell	*Trypanosoma cruzi*	HeLa	Stomach	Migration assay; IFI; FC	Maeda et al., 2016
Transwell	*Trypanosoma cruzi*	Caco-2; Vero; primary cardiac cells	Heart	ICC; cytotoxicity assay; absorption assay	Orlando et al., 2025
Transwell	*Trypanosoma cruzi*	Primary cardiac cells	Heart	Analysis of Chemokine/Cytokine; RT-qPCR; WB	Hernández et al., 2016

Transwell	*Cryptosporidium parvum*	Caco-2	Intestine	ICC; permeability assay; analysis of Chemokine/Cytokine; WB; RT-qPCR	Kumar et al., 2018
Transwell	*Plasmodium falciparum*	B cells	Blood	FC; analysis of Chemokine/Cytokine; ICC;	Reddy et al., 2021
Transwell	*Plasmodium falciparum*	BeWo; neutrophils	Blood	Migration assay; ICC;	Boström et al., 2017
Transwell	*Plasmodium berghei*	CD8+ T cells; primary brain cells	Brain	ICC; TEM; FC; scRNA-Seq analysis; qPCR	Wang et al., 2025
Spheroids/organoids	*Cryptosporidium parvum*	HIE; MIE	Intestine	TEER; scaffolds; ICC	Bhalchandra et al., 2020
Spheroids/organoids	*Cryptosporidium parvum*	iPSCs; LGR5-eGFP+ intestinal stem cells; ODMs	Intestine	qPCR; TEM; SEM; scRNA-seq	Heo et al., 2018
Spheroids/organoids	*Cryptosporidium parvum*	iPSCs	Lung	qPCR; IFI; ICC; TEM	Heo et al., 2018
Spheroids/organoids	*Cryptosporidium parvum*	MIE	Intestine	IFI; qPCR; senescence assays	Zhang, X. T. et al., 2016
Spheroids/organoids	*Trypanosoma cruzi*	HeLa	Human tissues	ICC	Rodríguez et al., 2019
Spheroids/organoids	*Trypanosoma brucei*	iPSCs	Brain	Transcriptomics; IHC	Chandrasegaran et al., 2023
Spheroids/organoids	*Trypanosoma cruzi*	Cardiac cells	Heart	ICC; qPCR; WB	Ferrão et al., 2018
Spheroids/organoids	*Trypanosoma cruzi*	Cardiac cells	Heart	TEM; ICC	Garzoni et al., 2008

Spheroids/organoids	*Trypanosoma cruzi*	Cardiac cells	Heart	Cytotoxicity assay; qPCR; ICC; analysis of Chemokine/Cytokine	de Almeida Fiuza et al., 2021
Spheroids/organoids	*Trypanosoma cruzi*	JEG-3; HBMECs; LLC-MK2	Placenta	FC; IFI	Silberstein et al., 2021
Spheroids/organoids	*Toxoplasma gondii*	Mouse intestinal model	Intestine	PCR; DIC; IFI	Martorelli Di Genova et al., 2019
Spheroids/organoids	*Toxoplasma gondii*	Pluripotent stem cells	Brain	ICC; TEM; transcriptomic	Seo et al., 2020
Spheroids/organoids	*Toxoplasma gondii*	ARPE-19	Retinal	qPCR; IFI; ICC; MORC	Cancela and colleagues (2025)
Spheroids/organoids	*Plasmodium falciparum*	hFLOs	Liver	Transcriptomics; ICC; TEM; qPCR	Yang et al., 2023
Spheroids/organoids	*Plasmodium vivax*	Human hepatocytes	Liver	SHG microscopy; functional assays	Chua et al., 2019
Scaffolds and biomaterials	*Leishmania major*	RAW, NKE	N/A	MTT	Bhattacharya et al., 2023
Scaffolds and biomaterials	*Acanthamoeba castellanii*	N/A	N/A	ICC; phase-contrast microscopy	Gutekunst et al., 2019
Microfluidics and organ-on-a-chip	*Trypanosoma brucei*	N/A	N/A	Optical tweezers microscope; ICC	Hochstetter et al., 2015
Microfluidics and organ-on-a-chip	*Giardia duodenalis*	N/A	N/A	Attachment assay	Lu et al., 2016
Microfluidics and organ-on-a-chip	*Giardia duodenalis*	C2BBe-1 cells (variant of Caco-2)	Intestine	Attachment assay; SEM	Hansen and Fletcher et al.,

					2008
Microfluidics and organ-on-a-chip	*Cryptosporidium parvum*	LGR5-eGFP+	Intestine	ICC; SEM; scRNA-seq	Nikolaev et al., 2020
Microfluidics and organ-on-a-chip	*Cryptosporidium parvum*	HCT-8	Intestine	qPCR; IFA; SEM	Gunasekera et al., 2024
Microfluidics and organ-on-a-chip	*Cryptosporidium hominis*	HCT-8	Intestine	qPCR; IFA; SEM; transcriptomics	Gunasekera et al., 2025
Microfluidics and organ-on-a-chip	*Entamoeba histolytica*	Caco-2	Intestine	ICC	Boquet-Pujadas et al., 2022
Microfluidics and organ-on-a-chip	*Toxoplasma gondii*	Caco-2; primary intestinal cells; HUVEC	Intestine; endothelium	ICC; qPCR; permeability assay	Humayn et al., 2022
Microfluidics and organ-on-a-chip	*Toxoplasma gondii*	hMVECs; primary neuron cells	Endothelium; nervous system	Migration assay; fluorescence microscopy	Kim et al., 2022
Microfluidics and organ-on-a-chip	*Plasmodium falciparum*	HUVEC; primary hepatocyte; primary splenocyte cells; RBCs	Liver; spleen; endothelium	Giemsa stain; optical microscopy; ICC	Rupar et al., 2023
Microfluidics and organ-on-a-chip	*Plasmodium falciparum*	HUVEC; primary hepatocyte; splenocyte cells; RBCs	Liver; spleen; endothelium	Giemsa stain; optical microscopy; ICC	Rupar et al., 2025
Microfluidics and organ-on-a-chip	*Plasmodium falciparum*	RBCs	Vasculature	Giemsa stain; optical microscopy; fluorescence microscopy	Shelby et al., 2003
Microfluidics and organ-on-a-chip	*Plasmodium falciparum*	RBCs	Vasculature	Hemolysis assay; flow cytometry; epifluorescence microscopy	Elizalde-Torrent et al., 2021

Microfluidics and organ-on-a-chip	*Plasmodium falciparum*	CHO; RAW; RBCs	Vasculature	Fluorescence microscopy; phagocytosis assay	Antia et al., 2007
Microfluidics and organ-on-a-chip	*Plasmodium falciparum*	BeWo; HUVEC; RBC	Placenta	Fluorescence microscopy; glucose measurement;	Mosavati, Oleinikov and Du, 2022
